# Biotin-Labelled Clavulanic Acid to Identify Proteins Target for Haptenation in Serum: Implications in Allergy Studies

**DOI:** 10.3389/fphar.2020.594755

**Published:** 2020-11-18

**Authors:** Ángela Martín-Serrano, Juan M. Gonzalez-Morena, Nekane Barbero, Adriana Ariza, Francisco J. Sánchez Gómez, Ezequiel Pérez-Inestrosa, Dolores Pérez-Sala, Maria J. Torres, María I. Montañez

**Affiliations:** ^1^ Allergy Research Group, Instituto de Investigación Biomédica de Málaga-IBIMA, Málaga, Spain; ^2^ Centro Andaluz de Nanomedicina y Biotecnología-BIONAND, Málaga, Spain; ^3^ Department of Structural and Chemical Biology, Centro de Investigaciones Biológicas Margarita Salas (CSIC), Madrid, Spain; ^4^ Department Química Orgánica, Universidad de Málaga-IBIMA, Málaga, Spain; ^5^ Allergy Unit, Hospital Regional Universitario de Málaga, Málaga, Spain; ^6^ Department of Medicina, Universidad de Málaga, Málaga, Spain

**Keywords:** betalactam, biotin tag, biotinylation, clavulanate, drug allergy, haptenation

## Abstract

Clavulanic acid (CLV) and amoxicillin, frequently administered in combination, can be independently involved in allergic reactions. Protein haptenation with β-lactams is considered necessary to activate the immune system. The aim of this study was to assess the suitability of biotinylated analogues of CLV as probes to study protein haptenation by this β-lactam. Two synthetic approaches afforded the labeling of CLV through esterification of its carboxylic group with a biotin moiety, via either direct binding (CLV-B) or tetraethylenglycol linker (CLV-TEG-B). The second analogue offered advantages as solubility in aqueous solution and potential lower steric hindrance for both intended interactions, with the protein and with avidin. NMR reactivity studies showed that both CLV and CLV-TEG-B reacts through β-lactam ring opening by aliphatic amino nitrogen, however with different stability of resulting conjugates. Unlike CLV conjugates, that promoted the decomposition of clavulanate fragment, the conjugates obtained with the CLV-TEG-B remained linked, as a whole structure including biotin, to nucleophile and showed a better stability. This was a desired key feature to allow CLV-TEG-B conjugated protein detection at great sensitivity. We have used biotin detection and mass spectrometry (MS) to detect the haptenation of human serum albumin (HSA) and human serum proteins. MS of conjugates showed that HSA could be modified by CLV-TEG-B. Remarkably, HSA preincubation with CLV excess only reduced moderately the incorporation of CLV-TEG-B, which could be attributed to different protein interferences. The CLV-TEG-B fragment with opened β-lactam was detected bound to the ^404–430^HSA peptide of the treated protein. Incubation of human serum with CLV-TEG-B resulted in the haptenation of several proteins that were identified by 2D-electrophoresis and peptide mass fingerprinting as HSA, haptoglobin, and heavy and light chains of immunoglobulins. Taken together, our results show that tagged-CLV keeps some of the CLV features. Moreover, although we observe a different behavior in the conjugate stability and in the site of protein modification, the similar reactivity indicates that it could constitute a valuable tool to identify protein targets for haptenation by CLV with high sensitivity to get insights into the activation of the immune system by CLV and mechanisms involved in β-lactams allergy.

## Introduction

β-lactam antibiotics are the second most consumed drugs and the most frequent ones eliciting allergic reactions (Doña et al., 2012). This poses an important clinical problem since, in the most severe cases, allergic reactions may be life-threatening and reduce the therapeutic options against infections. The frequency of allergic reactions associated with each drug varies over time according to consumption patterns (Martin-Serrano, 2018). Hence, currently, amoxicillin (AX) is the antibiotic most frequently eliciting immediate (IgE-mediated) allergic reactions (Fernandez et al., 2017). In addition, AX is nowadays frequently administered in combination with clavulanic acid (CLV), a β-lactam compound which inhibits β-lactamases activity (Torres et al., 2016). As a consequence of the increase in the frequency of AX-CLV administration, selective reactions to CLV are on the rise, reaching 30% of the allergic reactions induced by this combination (Torres et al., 2010; Blanca-Lopez et al., 2015; Fernandez et al., 2017; Salas et al., 2018).

Diagnosis of a suspected reaction after AX-CLV intake is a challenge, as it involves determining which of the two drugs is the responsible one (Torres et al., 2016). Skin test to CLV is only available in some countries and its sensitivity is not optimal. Therefore, drug provocation tests must be performed indirectly using both AX and AX-CLV to establish a diagnosis of CLV allergy (Doña et al., 2019). However, these *in vivo* tests are contraindicated in severe life threatening reactions. The ideal alternative is the performance of risk-free *in vitro* tests (Mayorga et al., 2016), although immunoassays for quantifying IgE specific to CLV are not available and have never been reported. Only *in vitro* tests such as basophil activation tests or histamine release tests have been used for the evaluation of patients (Torres et al., 2010; Pineda et al., 2015; Salas et al., 2018; Barbero et al., 2019). These functional assays use the CLV molecule to evaluate whether the drug induces cellular activation, but they show suboptimal sensitivity (Ariza et al., 2016b; Doña et al., 2017).

In the context of IgE-mediated reactions to β-lactams, drugs behave as haptens as they are assumed to covalently bind to carrier proteins to induce an immunological response (Ariza et al., 2011; Gonzalez-Morena et al., 2016). Both, the resulting structure of the conjugated drug (antigenic determinant) and part of the protein to which it is attached may be involved in the IgE recognition process (Ariza et al., 2015; Ariza et al., 2016a; Martín-Serrano et al., 2016). Developing new approaches for diagnosing CLV allergy and improving the existing ones requires the inclusion of CLV derivative structures recognized by the immune system, whose identification is much more complex compared with other β-lactam drugs. Complex reactivity of CLV and instability after protein conjugation have delayed the isolation and characterisation of the main CLV antigenic determinants (Barbero et al., 2019), and the lack of monoclonal antibodies against CLV has impeded the identification of proteins involved. Therefore, elucidating the mechanisms and structures involved in the immune system activation by CLV is required to advance in diagnosis.

Protein haptenation by CLV is assumed to occur similarly to other β-lactams (Edwards et al., 1988; Barbero et al., 2019), i.e., benzylpenicillin and AX, by nucleophilic opening of the β-lactam ring by protein amino groups from lysine residues (Batchelor et al., 1965; Yvon et al., 1990; Garzon et al., 2014). However, unlike penicillins, which render stable penicilloyl determinants, the resulting acylated structure of CLV is unstable and degrades, leading to small and heterogeneous epitopes with a very low density in the carrier (Edwards et al., 1988; Torres et al., 2016). We have recently reported the identification of a CLV determinant: N-protein, 3-oxopropanamide, which was addressed through a synthetic approach of its analogues and their ability to activate basophils in a higher proportion of patients compared with the native CLV (Barbero et al., 2019). Moreover, the same CLV fragments bound to protein were identified by proteomic approaches (Barbero et al., 2019). Based on its extraordinary ligand-binding capacity, human serum albumin (HSA) has been traditionally considered the main target protein in the haptenation process for β-lactams, and most studies have focused on characterisation of the penicilloyl-HSA conjugates (Jenkins et al., 2009; Martin et al., 2010; Whitaker et al., 2011; Garzon et al., 2014; Meng et al., 2016; Azoury et al., 2018). Regarding CLV, only a couple of recent studies have reported HSA haptenation, identifying stable N-protein, 3-oxopropanamide determinant on lysine residues in *in vitro* conjugation at physiological pH (Barbero et al., 2019) and in patients treated with the drug (Meng et al., 2016), whereas direct binding of CLV to lysine residues, subsequent degradation products, pyrazine conjugates and cross-linking conjugates were identified at high concentrations *in vitro* (Meng et al., 2016).

Besides HSA, other proteins can be target of haptenation with β-lactams. Transferrin (Magi et al., 1995; Ariza et al., 2012; Garzon et al., 2014) and immunoglobulins (light and heavy chains) (Ariza et al., 2012; Garzon et al., 2014) have been reported to be target serum proteins to be modified *in vitro* by ampicillin and/or AX. Besides serum proteins, surface and intracellular proteins have been reported to form antigenic determinants with β-lactams (Binderup and Arrigoni-Martelli, 1979; Watanabe et al., 1986; Watanabe et al., 1987; O´Donnell et al., 1991; Gonzalez-Morena et al., 2016; Sánchez-Gómez et al., 2017) and the intracellular haptenation process has been suggested to be cell type-dependent (Ariza et al., 2014). The identification of novel drugs or reactive metabolite conjugates with proteins is extremely challenging and relevant due to the role of these structures in the activation of the immune system (Labenski et al., 2009). The gold standard to detect and identify drug-protein conjugates is high resolution mass spectrometry (Garzon et al., 2014); nevertheless this approach requires sophisticated equipment that is not available to every laboratory. Alternatively, specific antibodies can be used through immunoassays (ELISA, immunoblotting) for the detection of these conjugates (Martin-Serrano, 2018); however, the lack of specific antibodies for some epitopes and the lack of sensitivity for the detection of low concentrations of conjugates or low degree of protein modification is the major drawback of these detection techniques (Tailor et al., 2016). The use of labeled drugs or reactive metabolites to enrich the drug modified fraction in complex samples and to improve the detection is an option to be considered (Koizumi et al., 2011; Ariza et al., 2014; Gonzalez-Morena et al., 2016). The avidin-biotin interaction provides great affinity and sensitivity, as well as the possibility of coupling modification of proteins by biotinylated compounds with several methods for detection, purification, and imaging (Ariza et al., 2014; Martin-Serrano, 2018). Previous studies have combined biotin labeling with proteomic techniques for the identification of potential protein targets for haptenation (Garzón et al., 2010; Ariza et al., 2014; Sánchez-Gómez et al., 2017; Shan et al., 2017) or modified protein residues (Havelund et al., 2017). The use of biotinylated AX (AX-B) has been shown to increase the detection sensitivity of AX modified serum proteins by immunological methods (Ariza et al., 2014) and has allowed the detection of novel cellular targets (Sánchez-Gómez et al., 2017).

Unlike AX, no antibody specifically targeting CLV is available, which has hampered the immunological detection of protein-CLV conjugates. To overcome this issue, in this work we envisaged an alternative strategy consisting in the design of appropriate biotinylated derivatives of CLV as highly sensitive and straightforward tools to study haptenation and developing methods to identify CLV target proteins. Two different structures, CLV-Biotin (CLV-B) and CLV-tetraethylenglycol-Biotin (CLV-TEG-B), were synthesized as probes for detecting haptenated serum proteins through streptavidin-based amplification technique. Both compounds bear biotin moiety linked through CLV carboxylic group without altering β-lactam group, which is reactive against proteins. The reactivity of biotinylated analogs of CLV and their ability to form conjugates was studied with a simple nitrogen nucleophile, as well as with HSA as protein model for haptenation. In addition, conjugation to a simple model peptide was analyzed, choosing ^182–195^HSA peptide due to its previous identification as target of AX (Garzon et al., 2014) and CLV (Barbero et al., 2019). The biotinylated derivative was demonstrated to be a straightforward tool to identify serum proteins target of modification.

## Materials and Methods

### Chemical Synthesis

#### Synthesis of Clavulanate-Biotin

##### Methyl Biotinate

The reported procedure (Martin-Serrano, 2018) was followed and adapted (Soares da Costa et al., 2012). Thionyl chloride (45 µL) was added slowly to a suspension of biotin (50 mg, 0.204 mmol) in methanol (1.2 mL) and the mixture was stirred at room temperature for 1 h. The reaction mixture was concentrated *in vacuo* to give quantitatively the methyl ester as a white solid (52 mg). Spectral data are in agreement with those reported in the literature (Tao et al., 2007).

##### Biotinol

The reported procedure(Martin-Serrano, 2018) was followed and adapted (Soares da Costa et al., 2012). To a suspension of methyl biotinate (52 mg, 0.204 mmol) in dry THF (2 mL) was added carefully LiAlH_4_ (31 mg, 0.816 mmol) and stirred at room temperature overnight. The reaction mixture was quenched with methanol (1 mL) and water (1 mL). MgSO_4_ was added to the mixture and it was stirred for additional 20 min. Then, the reaction mixture was concentrated *in vacuo*, filtered and washed with 1:4 MeOH/CH_2_Cl_2_ (10 mL). The filtrate was concentrated *in vacuo* to give quantitatively the target product as a white solid (47 mg). Spectral data are in agreement with those reported in the literature (Corona et al., 2006).

##### Biotin Tosylate

The reported procedure (Martin-Serrano, 2018) was followed and adapted (Soares da Costa et al., 2012). Tosyl chloride (47 mg, 0.245 mmol) was added to a suspension of biotinol (47 mg, 0.204 mmol) in dry pyridine (1.0 mL) in an ice bath. The reaction mixture was stirred at zero degrees for 1 h and at room temperature overnight. Then, it was diluted with CH_2_Cl_2_ (5 mL) and washed with aqueous HCl 1M (5 mL), aqueous saturated NaHCO_3_ (5 mL), water (5 mL), and brine (5 mL). The organic layer was dried over anhydrous MgSO_4_, filtered, concentrated *in vacuo* and purified by flash chromatography eluting with 5% methanol in CH_2_Cl_2_ to give a white solid (29%). Spectral data are in agreement with those reported in the literature (DeLaLuz et al., 1995).

##### Biotin Iodide

The reported procedure (Martin-Serrano, 2018) was followed (Iglesias-Sánchez et al., 2010). Biotin tosylate (75 mg, 0.196 mmol) and NaI (60 mg, 0.391 mmol) were stirred at reflux in acetone (10 mL) for 24 h. The solvent was removed under reduced pressure and the residue was dissolved in CH_2_Cl_2_ and the organic layer was successively washed with aqueous saturated sodium thiosulfate (10 mL) and water, dried over anhydrous Mg_2_SO_4_, and concentrated *in vacuo.* Purification of the crude material by flash chromatography eluting with 5% methanol in CH_2_Cl_2_ gave the target compound as a white solid (52 mg, 78%). Spectral data are in agreement with those reported in the literature (Iglesias-Sánchez et al., 2010).

##### 2-Biotin Clavulanate (CLV-B)

The reported procedure (Martin-Serrano, 2018) was followed and adapted (Brown et al., 1984). Commercially available potassium clavulanate (44 mg, 0.173 mmol) and previously synthesized biotin iodide (49 mg, 0.144 mmol) under nitrogen atmosphere were stirred in dry DMF (2 mL) at room temperature overnight. The solvent was removed *in vacuo* and the crude was purified by flash chromatography eluting with 5% methanol in CH_2_Cl_2_ to give the target compound as a white solid (35 mg, 60%).

^1^H-NMR (400 MHz, DMSO-d_6_): δ 6.43 (1H, s, NH), 6.36 (1H, s, NH), 5.69 (1H, d, *J* = 2.7 Hz, H_5_), 5.18 (1H, s, H_2_), 4.73 (1H, t, *J* = 6.8 Hz, H_1_″), 4.30 (1H, t, *J* = 7.3 Hz, H_12_′), 4.14–3.94 (5H, m, H_8_′+ H_2_′ + H_2_″), 3.62 (1H, dd, *J* = 16.8, 2.7 Hz, H_6_, diastereotopic protons), 3.13–3.09 (2H, m, H_6_ + H_7_′), 2.82 (1H, dd, *J* = 12.5, 5.1 Hz, H_13_′, diastereotopic protons), 2.58 (1H, d, *J* = 12.5 Hz, H_13_′), 1.60–1.23 (8H, m, H_6_′ + H_5_′ + H_4_′ + H_3_′); ^13^C-NMR (400 MHz, DMSO-d_6_): δ 175.5 (C7), 167.2 (C1′), 162.7 (C10′), 150.4 (C3), 101.2 (C1′), 87.5 (C5), 65.5 (C2′), 61.0 (C8′), 69.9 (C2), 59.2 (C12′), 55.5 (C2″), 55.4 (C7’), 46.0 (C6), 28.14, 28.11, 27.7 (C5′, C4′, C3′), 25.2 (C6′). C13′ signal overlaps with DMSO-d6 solvent signal (see HSQC). HRMS (C_18_H_25_N_3_O_6_S-H, 410.1385), found: 410.1385.

#### Synthesis of Clavulanate-Tetraethylenglycol-Biotin

##### 2-(2-(2-(2-Iodoethoxy)ethoxy)ethoxy)ethyl Biotinate

The reported procedure (Martin-Serrano, 2018) was followed and adapted (Li et al., 2008). DMSO (5 mL) was added into a 25-mL schlenk containing biotin (244 mg, 1.0 mmol) under nitrogen atmosphere at room temperature. Then, NaH (44 mg, 1.1 mmol, 60% dispersion in mineral oil) was added under N_2_ atmosphere and the reaction mixture was allowed to stir for 10 min. Then, previously prepared 1-iodo-2-(2-(2-(2-iodoethoxy)ethoxy)ethoxy)ethane (I-TEG-I) (555 mg, 1.4 mmol) in DMSO (2 mL) was added to the reaction and the mixture was stirred at room temperature overnight. Then, saturated aqueous NH_4_Cl was added. Subsequently, it was extracted with EtOAc (3 × 10 mL) and the combined organic phases were dried over anhydrous MgSO_4_, filtered and concentrated *in vacuo*. The crude was purified by flash chromatography eluting with 5% methanol in CH_2_Cl_2_ to give the target compound as a white solid (184 mg, 35%).

^1^H-NMR (400 MHz, CDCl_3_): δ 6.29 (1H, s, NH), 5.83 (1H, s, NH), 4.38–4.35 (1H, m, H_8_), 4.18–4.15 (1H, m, H_4_), 4.10–4.07 (2H, m, H_6_′), 3.62 (2H, t, *J* = 6.7 Hz, H_12_′), 3.57 (2H, t, *J* = 4.9 Hz, H_7_′), 3.53 (s, 8H, H_8_′, H_9_′, H_10_′, H_11_′, H_12_′), 3.13 (2H, t, *J* = 7.0 Hz, H_13_′), 3.04–2.99 (1H, m, H_5_), 2.76 (1H, dd, *J* = 12.8, 5.0 Hz, H_7_, diastereotopic protons), 2.61 (1H, d, *J* = 12.7 Hz, H_7_), 2.24 (2H, t, *J* = 7.6 Hz, H_4_′), 1.64–1.48 (4H, m, H_1_′, H_3_′), 1.36–1.27 (2H, m, H_2_′). ^13^C-NMR (400 MHz, CDCl_3_): δ 173.6 (C5′), 164.0 (C2), 71.8, 70.5, 70.46, 70.40, 70.07, 69.04 (C7′, C8′, C9′, C10′, C11′, C12′), 63.3 (C6′), 61.8 (C8), 60.0 (C4), 55.5 (C5), 40.4 (C7),33.7 (C4′), 28.26, 28.11 (C2′, C3′), 24.6 (C1′), 3.1 (C13′). HRMS (C_18_H_31_IN_2_O_6_S + H, 531.1020), found: 531.1015.

##### 2-Tetraoxadodecane-Biotin Clavulanate (CLV-TEG-B)

The reported procedure (Martin-Serrano, 2018) was followed and adapted as follows (Brown et al., 1984). Commercially available potassium clavulanate (53 mg, 0.223 mmol) and previously synthesized 2-(2-(2-(2-Iodoethoxy)ethoxy)ethoxy)ethyl biotinate (130 mg, 0.246 mmol) under nitrogen atmosphere were stirred in dry DMF (2 mL) at room temperature overnight. The solvent was removed under vacuum and the crude was purified by flash chromatography eluting with 5% methanol in CH_2_Cl_2_ to give the target compound as colourless oil (20 mg, 15%).

^1^H-NMR (400 MHz, CDCl_3_): δ 5.90 (1H, s, NH), 5.68 (1H, d, *J* = 2.3 Hz, H_5_), 5.35 (1H, s, NH), 5.05 (1H, d, *J* = 1.1 Hz, H_2_), 4.97 (1H, dt, *J* = 6.9, 1.3 Hz, H_1_″), 4.51–4.47 (1H, m, H_16_′), 4.31–4.19 (7H, m, H_20_′, H_2_′, H_2″_, H_9_′), 3.71–3.58 (12H, m, H_3_′, H_4_′, H_5_′, H_6_′, H_7_′, H_8_′), 3.48 (1H, dd, *J* = 16.7, 2.8 Hz, H_6_, diastereotopic protons), 3.16–3.11 (1H, m, H_15_′), 3.08 (1H, d, *J* = 16.7 Hz, H_6_), 2.89 (1H, dd, *J* = 12.8, 5.0 Hz, H_21_′, diastereotopic protons), 2.74 (1H, d, *J* = 12.8 Hz, H_21_′), 2.36 (2H, t, *J* = 7.3 Hz, H_11_′), 1.74–1.63 (4H, m, H_12_′, H_14_′), 1.48–1.39 (2H, m, H_13_′). ^13^C-NMR (400 MHz, CDCl_3_): δ 174.8, 173.8, 167.3, 163.7 (C=O), 152.0 (C3), 101.0 (C1″“), 87.9 (C5), 70.8, 70.6, 70.4, 69.3, 69.8 (oxygenated chain), 63.51, 63.49 (C2′, C9′), 62.0 (C20′), 61.7 (oxygenated chain), 60.5, 60.2 (C2, C16′), 57.2 (C2″), 55.6 (C15′), 46.5 (C6), 40.6 (C21′), 33.9 (C11′), 28.4, 28.3 (C12′, C13′), 24.8 (C14′). HRMS (C_26_H_40_N_3_O_11_S + H, 602.2378), found: 602.2378.

### Stability Studies

Freshly prepared solutions of CLV or CLV-TEG-B 1:1, at 10 mM concentration, in deuterated PBS were placed in an NMR tube and incubated at 37°C. The reactions were monitored by ^1^H-NMR registration after 1, 16, and 40 h (Martin-Serrano, 2018).

### Evaluation of CLV and CLV-TEG-B Reactivity Toward Amino Nucleophiles

Mixture solutions of CLV or CLV-TEG-B 1:1 with butylamine, at 10 mM concentration for each species were prepared in deuterated PBS and incubated at 37°C. The reactions were monitored by ^1^H-NMR registration after 15 min, 1, 16 and 40 h (Martin-Serrano, 2018).

### *In vitro* Modification of HSA or Serum Proteins by CLV–B or CLV-TEG-B

HSA-CLV-TEG-B conjugates were prepared by incubation of HSA at 10 mg/mL (0.15 mM) in PBS for 16 h at 37°C with decreasing concentrations of CLV-B and CLV-TEG-B (15 mM, 1.5 and 0.15 mM) in PBS, at 1:100, 1:10 and 1:1 protein/drug molar ratio. They were purified by dialysis filtration using Amicon filters (Merck- Millipore) and analyzed by matrix-assisted laser desorption/ionization-time of flight (MALDI-TOF) mass spectrometry (MS).

In addition, each biotinylated derivative, CLV-B and CLV-TEG-B, were freshly dissolved in DMSO and PBS (pH 7.4), respectively. For modification by biotinylated CLV derivatives, HSA at 10 mg/mL (0.15 mM) in PBS was incubated for 16 h at 37°C with decreasing concentrations of CLV-B or CLV-TEG-B (90 mM–0.05 µM). Then, conjugates were purified by dialysis filtration using Amicon filters (Merck-Millipore). For modification of serum proteins, human serum from healthy donors was incubated with CLV-TEG-B freshly prepared in PBS (pH 7.4) at 0.03 mM for 16 h at 37°C. All conjugates were analyzed by sodium dodecyl sulfate-polyacrylamide gel electrophoresis (SDS-PAGE) followed by modification detection with streptavidin-horseradish peroxidase (HRP) and ECL (Martin-Serrano, 2018), as described below.

### *In Vitro* Modification of HSA Peptide by CLV-TEG-B

CLV-TEG-B was freshly prepared in PBS (pH 7.4) at 0.15 mM (Ariza et al., 2012) and incubated with 13.5 mM ^182–195^HSA peptide (1,518.68 Da) in PBS (pH 7.4) for 24 h at 37°C. Resulting conjugates were purified using PD G-10 Desalting Columns (GE Healthcare) and then analyzed by mass spectrometry (Martin-Serrano, 2018), as described below.

### Competition Between CLV and CLV-TEG-B for HSA Modification

HSA (10 mg/mL, 0.15 mM) was preincubated with CLV (80–96,000 µM) in PBS (pH 7.4) for 16 h at 37°C. After preincubation, CLV-B was added to each sample to a final concentration of 80 µM and then incubated for 2 h at 37°C. Resulting conjugates were than analyzed by SDS-PAGE and drug modification was detected by transfer to membrane and biotin detection with avidin-HRP (Martin-Serrano, 2018).

### CLV- Enrichment of Protein-Biotinylated Drug Fraction by Avidin Affinity Chromatography

Resulting samples of the incubation of proteins with biotinylated compounds were filtered and then purified using agarose beads coated with Neutravidin (Thermo Fisher Scientific). Samples were incubated with the beads for 2 h at room temperature and then resin was washed with 50 mM Tris-HCl pH 7.4, 1 mM EDTA, 1 mM EGTA, 1% NP-40 and 5% SDS buffer to eliminate unspecifically bound proteins (Garzón et al., 2010; Martin-Serrano, 2018). Finally, biotinylated fraction was released from the resin using a buffer containing SDS and β-mercaptoethanol. In parallel, control HSA was subjected to affinity purification to assay the possibility of unspecific retention with the resin (Garzón et al., 2010; Martin-Serrano, 2018).

### Mass Spectrometry Analysis of CLV-TEG-B- Modified HSA

The MALDI-TOF mass spectra of HSA protein and peptide modification with CLV-TEG-B were acquired at Central Service for Research Support (SCAI, University of Malaga), Proteomic Unit, using a MALDI TOF TOF Bruker UltraFlextreme (Martin-Serrano, 2018). Experiments were recorded by dissolving conjugates in milliQ water containing 0.1% trifluoroacetic acid (TFA) and using sinapinic acid (SPA) or α-cyano-4-hydroxycinamic acid (CHCA) as the matrix for proteins or peptides, respectively.

*In vitro* HSA modification with CLV-TEG-B (enriched biotinylated fraction prepared at 1:10 protein/drug molar ratio) was studied by liquid chromatography-mass spectrometric (LC-MS/MS) analysis, acquired at Proteomic Laboratory at National Center of Biotecnology (CNB,CSIC, Madrid). After sample digestion with trypsin, chymotrypsin and LysC, collision-induced dissociation (CID) fragmentation was performed using a TripleTOF 5600 Q-TOF mass spectrometer (SCIEX) for peptide sequencing and protein matching (MASCOT). The mass spectrometry proteomics data have been deposited to the ProteomeXchange Consortium via the PRIDE (Perez-Riverol et al., 2019) partner repository with the dataset identifier PXD021727.

### SDS-PAGE Electrophoresis and Biotinylation Detection by Blot Followed by Biotin Detection

Samples of HSA and human serum conjugates with CLV-B or CLV-TEG-B containing 2–4 µg of protein were separated in 12% SDS-PAGE. Then, proteins were transferred to a PVDF membrane (Trans-Blot Turbo Mini PVDF Transfer Packs, Bio-Rad) using Trans-Blot Turbo Transfer System from Bio-Rad following manufacturer’s indications. For biotin detection, blots were incubated with HRP-streptavidin (Amersham, GE Biosciences) at 1/1,000 dilution and ECL detection (Clarity Western ECL Substrate, Bio-Rad). Estimation of the biotinylation degree was made by comparison with a biotinylated BSA standard (Pierce) (Gharbi et al., 2007). To check HSA load, blots were previously incubated with anti-HSA primary antibody (Santa Cruz Biotechnology) at 1 μg/mL and polyclonal rabbit HRP-anti-mouse IgG (DAKO) at 1/2,000 dilution and then stripped with HCl guanidine 8 M before biotinylation detection. Chemiluminiscence was used for detection (Clarity Western ECL Substrate, Bio-Rad) and images were analyzed using ImageQuant LAS4000 (GE Healthcare) (Martin-Serrano, 2018). We analyzed images obtained with ImageJ software (National Institutes of Health) for three replicates and expressed the results as pmol biotin/pmol HSA.

### Two-Dimensional Electrophoresis and Protein Identification

For two-dimensional electrophoresis, samples were processed by a procedure similar to that previously described (Ariza et al., 2012) (Martin-Serrano, 2018). Aliquots of control and CLV-TEG-B-treated human serum (protein:drug ratio 1:0.192) containing 20 µg of protein were precipitated with cold acetone stirring for 16 h at 4°C. Then, acetone was decanted and the pellet completely dried using a SpeedVac system. The dried pellet was resuspended in 278.4 µL of IEF simple buffer (4% CHAPS, 2 M thiourea, 7 M urea, 100 mM DTT, and 0.4% Bio-lyte ampholytes). Sample was then divided in two aliquots and loaded on two ReadyStrip IPG Strips (pH 3–10 lineal, 7 cm, Bio-Rad) for isoelectric focusing on a Protean IEF cell (Bio-Rad), following the instructions of the manufacturer. Before the second dimension, strips were equilibrated in 6 M urea, 2% SDS, 0.375 M Tris-HCl pH 8.8, 20% glycerol and bromophenol blue containing 130 mM DTT for the first equilibration step and 135 M iodoacetamide for second step. Strips were then placed on top of duplicated 10% polyacrilamide SDS gels. One of the gels was subsequently transferred to a PVDF membrane and used for localization of proteins modified by CLV-TEG-B by biotin detection.

The duplicate gel was stained for total protein with Comassie staining and it was used for spot excising and identification at the CAI Técnicas Biológicas, Unidad de Proteómica, Facultad de Farmacia (Universidad Complutense de Madrid, Madrid, Spain). The spots of interest were then manually excised from gels. Proteins selected for analysis were in-gel reduced, alkylated and digested with trypsin according to previous literature (Sechi and Chait, 1998; Martin-Serrano, 2018). Briefly, the samples were reduced with 10 mM DTT in 25 mM ammonium bicarbonate for 30 min at 56°C and subsequently alkylated with 25 mM iodoacetamide in 25 mM ammonium bicarbonate for 15 min in the dark. Finally, samples were digested with 12.5 ng/μL sequencing grade trypsin (Roche Molecular Biochemicals) in 25 mM ammonium bicarbonate (pH 8.5) overnight at 37°C. After digestion, the supernatant was collected and 1 μL was spotted onto a MALDI target plate and allowed to air-dry at room temperature. Then, 0.6 μL of a 3 mg/mL of α-cyano-4-hydroxy-cinnamic acid matrix (Sigma) in 50% acetonitrile were added to the dried peptide digest spots and allowed again to air-dry at room temperature. MALDI-TOF MS analyses were performed in a 4800 Plus Proteomics Analyzer MALDI-TOF/TOF mass spectrometer (Applied Biosystems, MDS Sciex, Toronto, Canada). The MALDI-TOF/TOF operated in positive reflector mode with an accelerating voltage of 20,000 V. All mass spectra were calibrated internally using peptides from the auto digestion of trypsin. For protein identification SwissProt 20170116 (553222 sequences; 198133818 residues) with taxonomy restriction to human was searched using MASCOT 2.3 (www.matrixscience.com) through the software Global Protein Server v 3.6 (ABSciex). Search parameters were set as follows: enzyme, trypsin; fixed modifications, carbamidomethyl (C); variable modification, oxidized methionine; one missed cleavage allowed; peptide tolerance, 50 ppm. Probability scores greater than the score fixed by MASCOT were considered significant if *p* < 0.05.

The mass spectrometry proteomics data have been deposited to the ProteomeXchange Consortium via the PRIDE (Perez-Riverol et al., 2019) partner repository with the dataset identifier PXD021675.

## Results

### Synthesis and Characterisation of Biotinylated Clavulanic Acid (CLV-B and CLV-TEG-B)

Two different biotin derivatives of CLV were designed as probes for detecting haptenated serum proteins. Both approaches consisted in the successful labeling of CLV through its carboxylic group with a biotin moiety, via either direct binding or hydrophilic spacer linker, without affecting the CLV β-lactam ring.

**FIGURE 1:**
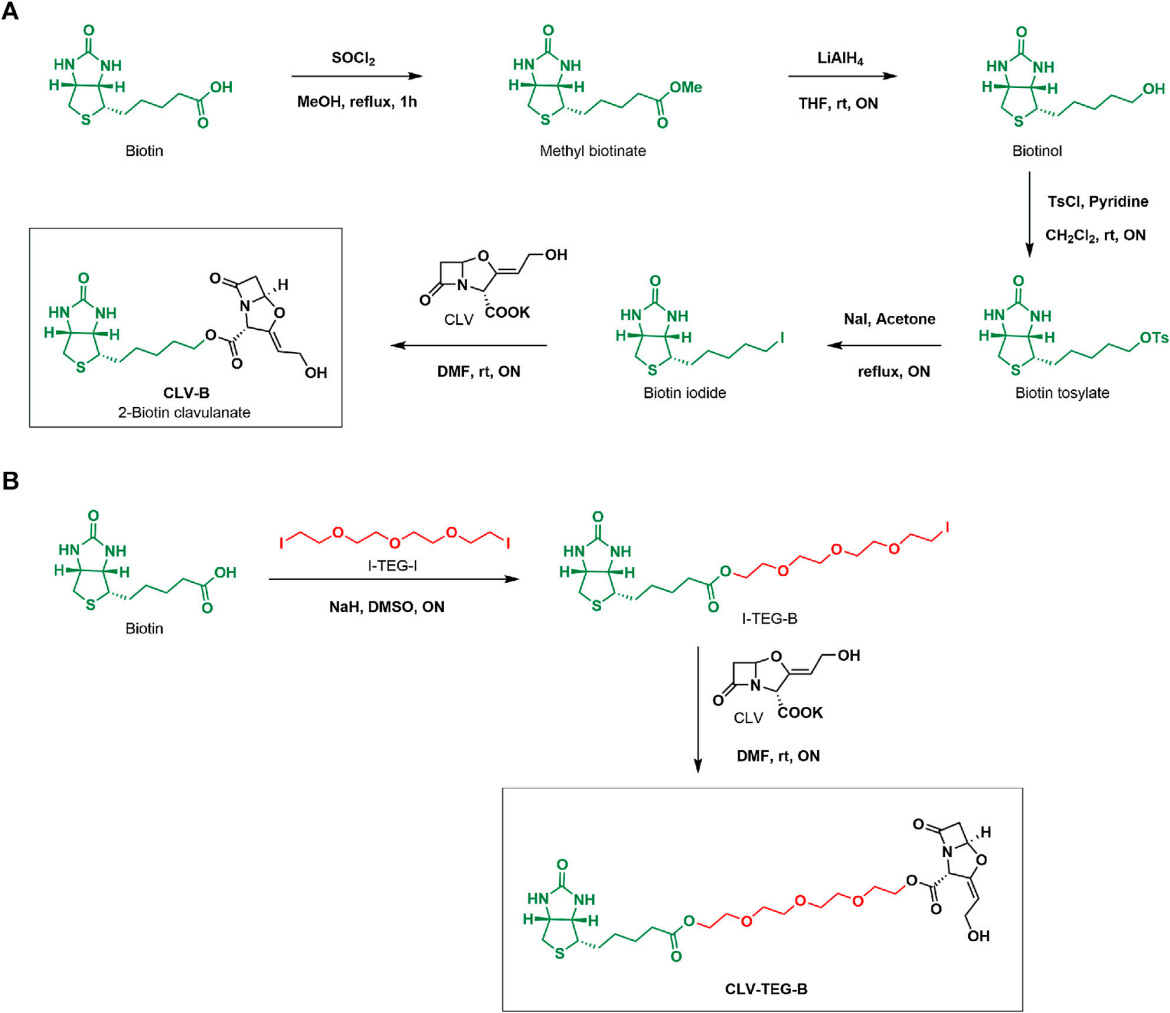
Synthesis of biotinylated derivatives of clavulanic acid (CLV). **(A)** CLV-B and **(B)** CLV-TEG-B. CH_2_Cl_2_, dichloromethane; DMF, dimethylformamide; DMSO, dimethyl sulfoxide; LiAlH_4_, lithium aluminum hydride; MeOH, methanol; NaI, sodium hydride; ON, overnight; rt, room temperature; SOCl_2_, thionyl chloride; THF, tetrahydrofurane; TsCl, tosyl chloride.

The first approach consisted in the straight labeling CLV resulting in CLV-B (Figure 1A). The synthetic sequence involved biotin esterification to form the methyl ester, further reduction to alcohol and subsequent tosylation that eventually allowed substitution by an iodide group. The resulting biotin iodide was used for esterification of CLV allowing biotinylation of the drug. The product was isolated with 73% average yield. The chemical structure of CLV-B and the synthetic intermediates were confirmed by conventional techniques of NMR and MS. The resulting compound was not completely soluble in water and DMSO was used to make it soluble in aqueous media for NMR characterisation (Supplementary Figures S1, S2) as well as for further protein incubation experiments.

The second approach employed an extending tetraethylenglycol (TEG) linker between CLV and the biotin moiety by means of a two-step synthetic process (Figure 1B). First, hydroxyl groups from TEG chain were tosylated and further substituted by iodide. Esterification reaction of biotin with TEG di-iodide (I-TEG-I) afforded biotin-TEG-iodide (I-TEG-B), which was used for the esterification of CLV leading to the target molecule CLV-TEG-B, isolated with 62% average yield. The hydrophilic TEG linker provides solubility to the compound in aqueous media. Reactions were monitored using ^1^H-NMR (Supplementary Figure S3) to confirm the completion of reactions. Final and intermediate products were appropriately purified and chemical structures were confirmed by conventional NMR (Supplementary Figures S4, S5) and MS techniques. This second derivative was completely soluble in water. Since aqueous solubility was a valuable property to perform following protein modification experiments, we mainly focused on CLV-TEG-B further on.

### Stability and Reactivity of CLV and CLV-TEG-B

Due to their similar structures it is expected that both biotinylated compounds, i.e., CLV-B and CLV-TEG-B, present similar stability and reactivity. The main difference between both molecules is the presence of the PEG spacer that confers water solubility to CLV-TEG-B, which are the optimal conditions to evaluate stability and reactivity for further protein experiments.

We compared both the stability of CLV and CLV-TEG-B at neutral pH and physiological salt concentration and their acylation reactivity toward nitrogen nucleophile at NMR scale. Proton chemical shifts for H5 and H6 in the β-lactam ring were used to monitor these processes.

We performed stability studies of CLV-TEG-B dissolved in deuterated PBS (Supplementary Figure S6). Spectra revealed that after 1 h of incubation 20% of the β-lactam ring was opened and kept on opening over time, with 50% of opened β-lactam at 16 h and 58% at 40 h. In addition, after 1 h, the peak corresponding to the oxazolidine ring (signal 1′) seems to be affected, which could indicate some kind of degradation (as oxazolidine ring opening or double bond isomerisation). Spectra were analyzed to compare the stability between CLV and CLV-TEG-B in deuterated PBS, at neutral pH and physiological salt concentration, showing that CLV is more stable than CLV-TEG-B since β-lactam opening did not take place for the former (Supplementary Figure S7).

**FIGURE 2:**
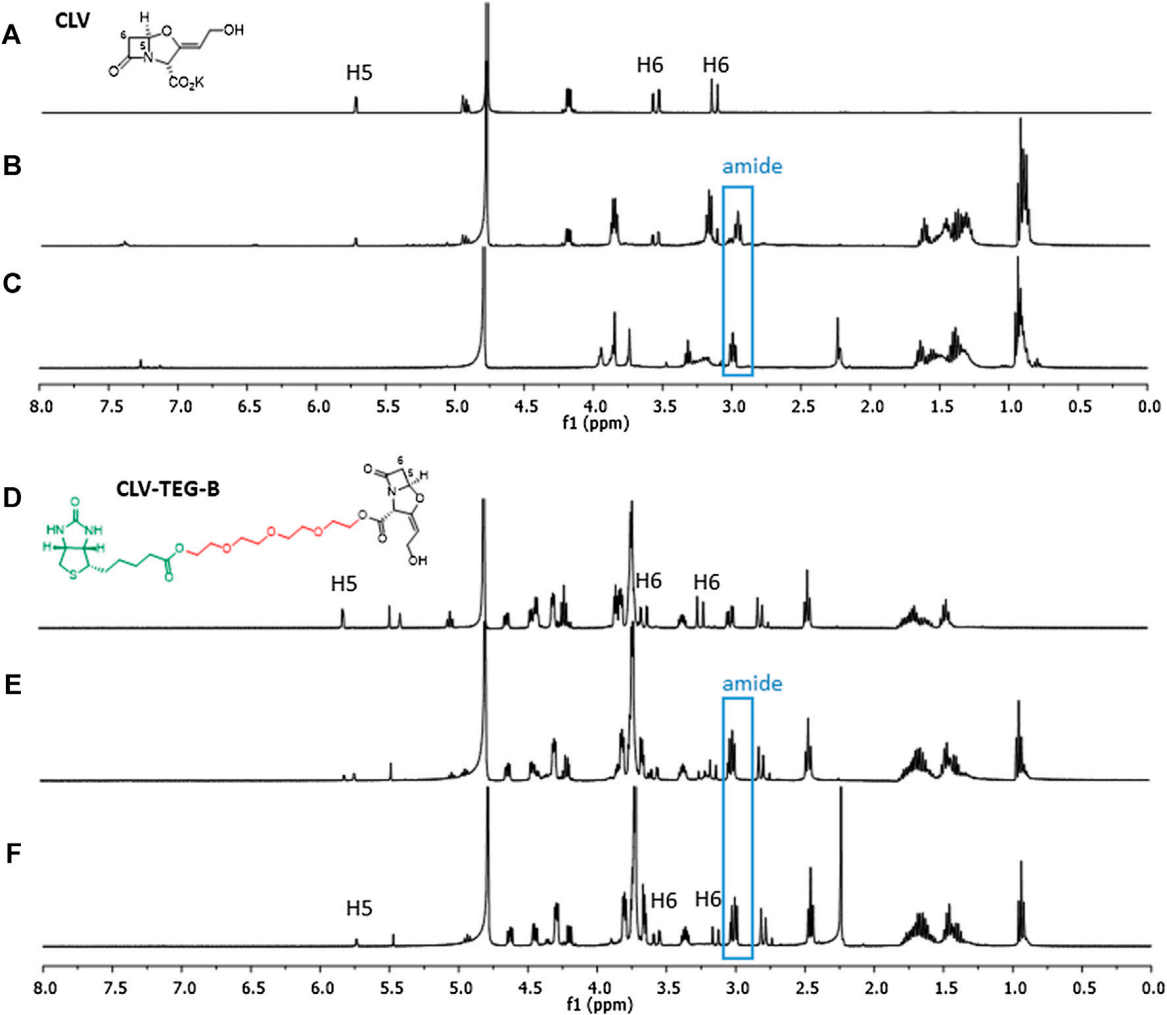
Reactivity of clavulanic acid (CLV) and CLV-tetraethylenglycol-Biotin (CLV-TEG-B). ^1^H NMR of each compound at similar conditions: **(A)** CLV control and **(D)** CLV-TEG-B control, after 15 min incubation in deuterated PBS. **(B)** CLV and **(E)** CLV-TEG-B, incubated with 1 equivalent of butylamine for 15 min **(C)** CLV and **(F)** CLV-TEG-B, incubated with 1 equivalent of butylamine for 16 h min. After addition of butylamine is observed amide formation for both compounds. Subsequently, degradation occurs in the case of CLV and shifted signals for protons corresponding to β-lactam (H5 and H6) are observed in the case of CLV-TEG-B.

The acylation abilities of CLV-TEG-B and CLV were studied toward butylamine, a simple nitrogen nucleophile molecule that mimics lateral chain of lysine residues, and therefore can conjugate to β-lactam (Figure 2). Results of CLV-TEG-B reactivity studies showed that after 15 min incubation with 1 equivalent of butylamine there was no remaining signal corresponding to methylene protons next to the amine in butylamine at 2.6 ppm and the appearance of a triplet at 3.2 ppm, both consistent with amide bond formation (Supplementary Figure S8). A shift in signals corresponding to β-lactam protons (H5 and H6) was observed after 15 min of incubation and the shift was complete after 16 h (see changes in signals H5 and H6), which could evidence β-lactam ring opening and/or other kind of ring modification. Spectra of CLV incubated with butylamine for 15 min showed that 60% of butylamine was forming amide and 40% remained as butylamine, as deduced by integration of signals corresponding to methylene protons closest to the butylamine nitrogen (Supplementary Figure S9). Moreover, the percentage of CLV conjugated to butylamine seems to degrade into other compounds whose structures cannot be elucidated with obtained data. Only a decreased integration of original shifts of CLV can be observed, consistent with the percentage of amide formed, however discerning where signals shift is not clear, unlike in the case of CLV-TEG-B.

### Characterisation of HSA-CLV-TEG-B Conjugates by MALDI-TOF and LC-MS

To confirm that conjugation takes place with proteins similarly with CLV and CLV-TEG-B, we used CLV- and CLV-TEG-B treated-HSA as model and analyzed the conjugates using proteomics. First MALDI-TOF MS techniques showed that incubation of HSA in the presence of CLV-TEG-B at 1:100, 1:10 and 1:1 M ratio caused an increase in the mass of the protein, which indicates incorporation of the drug derivative. (Supplementary Figures S10–S13). MS spectra of the dialyzed conjugates shows a peak at 66,467, 66,596 and 68,091 Da, shifted by 35, 164 and 1,659 Da with regard to the HSA control sample, for 1:100, 1:10 and 1:1 respectively, indicating a modification dependent of the biotinylated drug concentration, as previously reported with CLV treated-HSA (Barbero et al., 2019). The shift of the peak correspond to an average of different species as no homogeneous haptenation of the HSA may happen, and therefore we could not confirm neither the number of CLV derivatives bound to the protein nor the molecular weight of the added fragments.

To study in depth HSA residues modified by CLV-TEG-B and compared with previous results obtained with CLV (Barbero et al., 2019), HSA-CLV-TEG-B 1:10 conjugate was analyzed on a SCIEX 5600 TripleTOF spectrometer with CID fragmentation. The attempts to analyze the dialyzed conjugate were unsuccessful, however we observed that for the enriched biotinylated conjugate digested with chymotrypsin, a peptide modified with a mass increment of 600.2 Da was found (Supplementary Figures S14, S15): ^404–430^HSA (QNALLVRYTKKVPQVSTPTLVEVSRNL). Both MS1, showing the m/z value corresponding to the parental ion (Supplementary Figure S14) and MS2 spectra (Supplementary Figure S15), are fully compatible with this modified ^404–430^HSA peptide. The fragmentation spectrum (Supplementary Figure S15) shows interesting information corresponding to the y2, y3, y4, y5, y6, y7, y8, y9, y10 and y11 ion fragments, as well as b1 and b6 ions, in addition to some secondary fragments. The involved residue cannot be identified based on these ion series, although they suggest the discarding the TPTLVEVSRNL and QNALLV fragments, and therefore the addition could be in any nucleophilic aminoacid of the RYTKKVPQVS fragment.

The mass increment (600.2 Da) is consistent with the *in vitro* CLV-TEG-B covalent binding to HSA, by the β-lactam opening. In order to characterize the modification in a simpler model, an HSA peptide containing several lysine residues, ^182–195^HSA peptide (LDELRDEGKASSAK), previously identified as target of AX (Garzon et al., 2014) and CLV (Barbero et al., 2019), was incubated with a 90 M excess of CLV-TEG-B and analyzed by MALDI-TOF MS (Supplementary Figure S16). Spectra show that only a low proportion of peptide was modified, but the mass increment (600.2 Da) is in agreement with the previous approach, indicating the coupling of one moiety of CLV-TEG-B.

### Detection of HSA-Biotinylated CLV Conjugates by Blot and Biotin Detection

**FIGURE 3:**
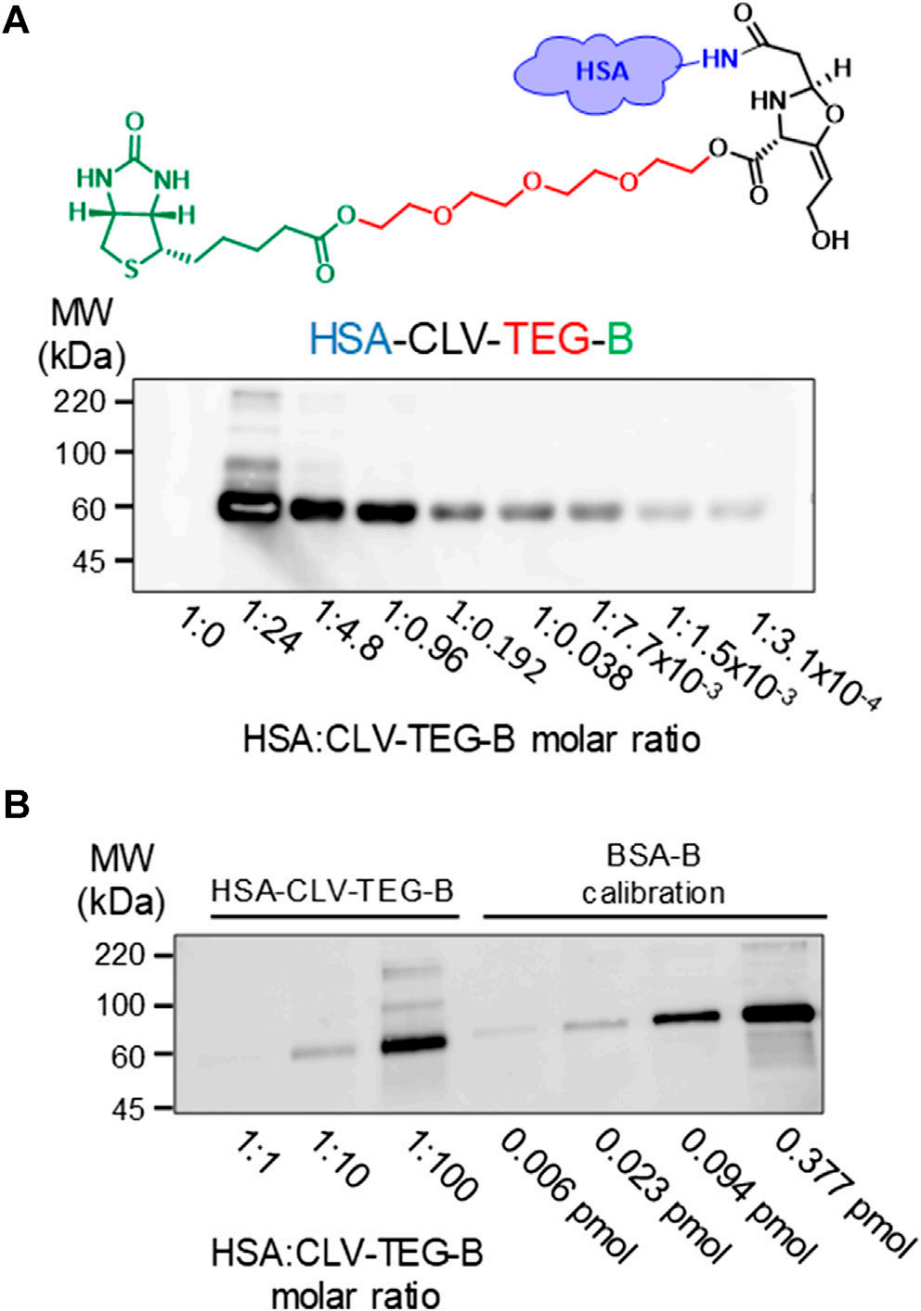
Detection of modification of HSA by CLV-tetraethylenglycol-Biotin (CLV-TEG-B). Human serum albumin (HSA) was incubated in the presence of CLV-TEG-B for 16 h at 37°C. HSA:CLV-TEG-B molar ratios are shown in figure **(A)** Aliquots of the haptenation reactions containing 2 µg of protein were analyzed by SDS-PAGE and CLV-TEG-B modification was detected by blot with streptavidin-HRP and ECL detection **(B)** 0.15 µg aliquots of HSA-CLV-TEG-B **(left)** and 0.006–0.377 pmol aliquots of BSA-B standards **(right)** were analyzed by SDS-PAGE followed by blot with streptavidin-HRP and ECL detection.

We analyzed the ability of biotinylated derivatives of CLV to bind covalently to proteins and their usefulness for the detection of CLV-protein conjugates by blot followed by biotin detection, since no anti-CLV antibodies are available. We incubated HSA with increasing concentrations of biotinylated derivatives of CLV in PBS (pH 7.4) (protein/biotinylated CLV ratios from 1:3.07 × 10^−4^ to 1:600) Results showed that both biotinylated derivatives of CLV bind covalently to HSA and that protein modification was dose-dependent. Signal detected by blot and detection with avidin-HRP was specific for biotinylated derivatives bound on HSA since no signal was detected for control HSA, and it was detected even at very low protein/biotinylated ratio, which confirms the high sensitivity of the method. A similar haptenation pattern was obtained for both biotinylated derivatives, and CLV-TEG-B was selected for performing further studies due its better water solubility properties compared with CLV-B (Supplementary Figure S17). Then, a more detailed dose-response analysis was performed with HSA-CLV-TEG-B conjugates, confirming their usefulness in the detection of HSA conjugates with high sensitivity (Figure 3A). The degree of incorporation of CLV-TEG-B was estimated by comparison with a biotinylated BSA standard and results representative of three assays showed values of 0.0935; 0.013; 0.00055 pmol biotin/pmol HSA for molar ratios of HSA:CLV-TEG-B 1:100; 1:10 and 1:1, respectively (Figure 3B). These results suggest that the extent of protein modification is not complete. Since the presence of non-conjugated protein could interfere in further detection analysis of the drug-protein conjugate, enrichment of the fraction of modified proteins would improve the characterisation and identification of conjugates. Protein fractions purified on neutravidin-agarose were analyzed by SDS-PAGE, and as it is shown in Supplementary Figure S18, the enrichment of HSA-CLV-TEG-B fraction increase the signal detection.

### CLV and CLV-TEG-B Competition for Protein Binding Sites

**FIGURE 4:**
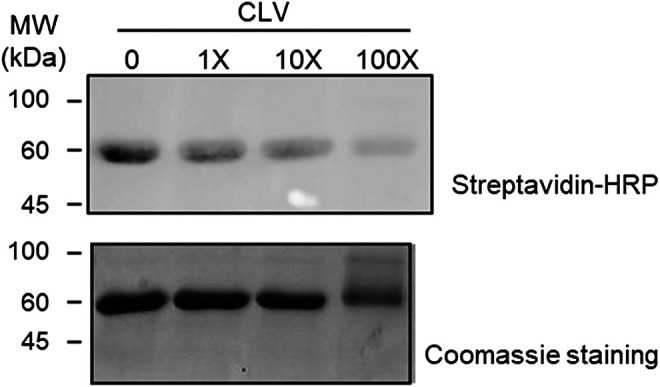
Competition assay between clavulanic acid (CLV) and CLV-tetraethylenglycol-Biotin (CLV-TEG-B). Human serum albumin (HSA) was preincubated with increasing concentrations of CLV (80, 800 and 8,000 μM) for 16 h at 37°C and then with a fixed concentration of CLV-TEG-B (80 μM) for 2 h at 37°C. Resulting adducts were analyzed by SDS-PAGE and CLV-TEG-B modification was detected by blot with streptavidin-HRP and ECL detection **(top panel)**. Total protein was detected by Coomassie staining **(bottom panel)**.

In order to compare the binding capacity of both structures, competition assays were performed. HSA was incubated for 2 h with 80 µM CLV-TEG-B, after preincubation (16 h) with increasing concentrations of CLV. Results showed that the preincubation of HSA in the presence of an excess of CLV reduced the formation of conjugates containing CLV-TEG-B (Figure 4), which may indicate that both compounds may bind on HSA common binding sites. Incubations using the highest concentration of CLV resulted in protein aggregation, which could be explained by the formation of cross-linking conjugates that has been reported at high CLV concentration *in vitro* (Meng et al., 2016). This is similar to the observed behavior in HSA incubations with high concentrations of AX (Ariza et al., 2012).

### Detection of CLV-TEG-B Candidate Target Proteins in Human Serum

**FIGURE 5:**
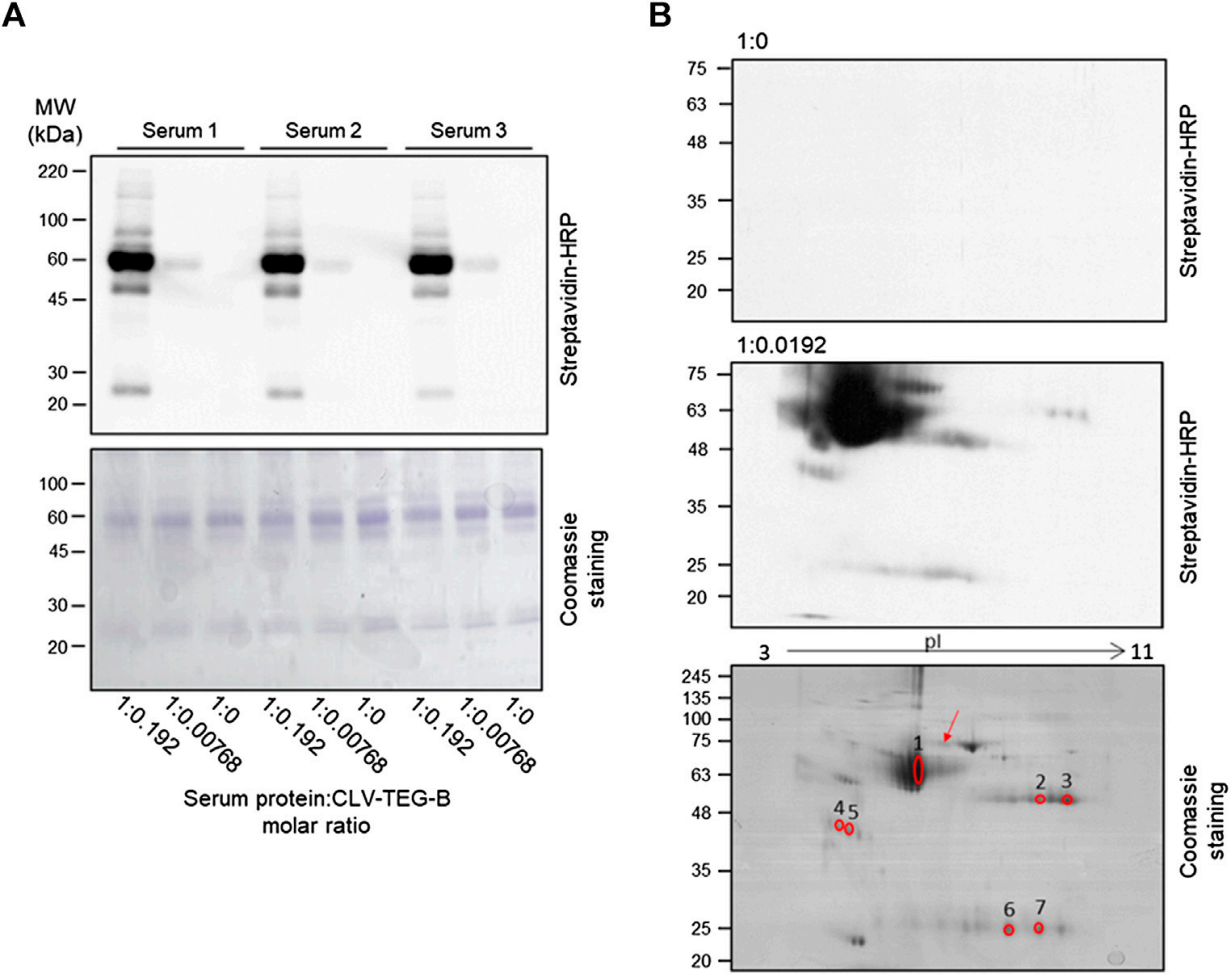
Detection of modification of serum proteins by clavulanic acid (CLV)-tetraethylenglycol-Biotin (CLV-TEG-B) and identification of targets. Human sera were incubated in the presence of CLV-TEG-B for 16 h at 37°C. **(A)** 4 µg aliquots of resulting adducts were analyzed by SDS-PAGE and CLV-TEG-B modification was detected by blot with streptavidin-HRP and ECL detection. Lower panel shows the Coomassie staining for total proteins visualization. **(B)** 50 µg aliquots of resulting adducts were subjected to 2D-eletrophoresis on duplicate gels, after which, one of the gels was used for detection of modified proteins by transfer to membrane and biotin detection with avidin-HRP and the other one was used for protein staining with Coomassie. Matched spots were excised from the gel and used for tryptic digestion and peptide fingerprint analysis. The blot in the middle panel is deliberately overexposed in order to show the signals corresponding to modified immunoglobulin chains. The red arrow point towards a spot that could be proposed as transferrin, on the bases of its molecular weight and isoelectric point.

In addition to HSA, other plasma proteins may be covalently modified by CLV-TEG-B and, to analyze that possibility, we incubated human serum with CLV-TEG-B and modified proteins were detected by transfer to membrane and biotin detection with avidin-HRP. Monodimensional SDS-PAGE allowed the observation of multiple positive bands, even with the lowest concentration of CLV-TEG-B used (Figure 5A), confirming that besides HSA, other serum proteins were target of haptenation. In order to identify the target proteins, samples were analyzed by two-dimensional electrophoresis followed by peptide fingerprint analysis by tryptic digestion and MALDI-TOF MS (Figure 5B; Table 1). Identified proteins included HSA, heavy and light immunoglobulin chains and haptoglobin. Although not included in this analysis, the haptenation of transferrin could be proposed on the bases of its molecular weight and isoelectric point in 2D gels.

**TABLE 1:** Identification of human serum proteins as targets for haptenation by CLV-tetraethylenglycol-Biotin by mass spectrometry. Data under superscripts c through h are from MASCOT.

Spot number^a^	Accession code^b^	Protein name	Total score^c^	Limit score	MW (Da)^d^	pI^e^	Matched peptides^f^	Coverage (%)^g^
1	P02768	Human serum albumin	470	56	71,317	5.92	43	69
2	P01857	Ig gamma-1 chain C region human	119	56	36,596	8.46	13	52
3	P01857	Ig gamma-1 chain C region human	197	56	36,596	8.46	18	66
4	P00738	Haptoglobin human	128	56	45,861	6.13	18	35
5	P00738	Haptoglobin human	102	56	45,861	6.13	16	33
6	P01834	Ig kappa chain C region human	101	56	11,773	5.58	8	76
7	P01834	Ig kappa chain C region human	81	56	11,773	5.58	7	76

a Spot numbering as shown in 2-DE Coomassie gel in Figure 5.

b Protein accession code from NCBI database.

c Mascot total score.

d Theoretical molecular weight (Da).

e Theoretical pI.

f Number of matched peptides.

g Protein sequence coverage for the most probable candidate as provided by Mascot.

## Discussion

CLV is a potent inhibitor of β-lactamase enzyme of increasing interest. Due to the increasingly worrying problem of antibiotic resistance, its consumption is on the rise and, as a consequence, the number of reported selective allergic responses induced by CLV after AX-CLV intake has increased significantly (Fernandez et al., 2017; Montañez et al., 2017). Unfortunately, the diagnostic work-up aiming to identify which drug of the AX-CLV combination is responsible for a reaction is not trivial. Risky *in vivo* tests show suboptimal sensitivity for CLV due to the fact that testing must be performed with AX-CLV combination, in which CLV is always in a lower ratio. This impedes both discriminating the drug eliciting the reaction and reaching CLV concentrations high enough to trigger reactions (Fernandez et al., 2017). On the other hand, *in vitro* tests to diagnose IgE-mediated reactions to CLV are limited to the basophil activation test using native CLV, with 40–50% of sensitivity (Mayorga et al., 2019). However, a recent study has reported that the inclusion of synthetic determinants of CLV increased the sensitivity of this assay up to 69%. Interestingly, only determinants with a N-protein, 3-oxopropanamide structure and ability for protein conjugation (spontaneous reactivity against amino groups) were found to induce basophil activation (Barbero et al., 2019). This finding indicates that drug-protein conjugates play a crucial role in the induction of allergies. Therefore, after getting insight into potential determinants responsible for allergies to CLV, herein we investigate potential proteins targets of CLV haptenation. A convenient strategy would be to follow a procedure similar to that reported for immunological detection of AX–protein conjugates with antibodies recognizing the lateral chain of the AX molecule, which successfully allowed the identification of serum proteins coupled to AX (Ariza et al., 2012). However, the lack of a suitable antibody against CLV has prevented the use of immunological detection approaches for our objective. Therefore, we considered label-drug techniques. Despite the fact that label-dependent techniques cannot be used to study conjugate formation in patients and usually do not provide information on the site of modification and/or structure of the conjugates, they are quite useful to visualize conjugates and to identify the modified proteins (Gonzalez-Morena et al., 2016). This is a big step to gain insight into conjugates formation in cases in which there are no specific antibodies against the drug or the conjugation takes place in such a low extension that it hampers detection using label-free approaches.

With the aim of detecting serum proteins that conjugate to CLV, biotin was chosen as a tag due to its extremely high affinity for streptavidin and the lack of impact on the properties of its substrate in most cases (Diamandis and Christopoulos, 1991). The strength of the biotin-streptavidin interaction makes this approach very sensitive. However, in order to retain their proper biological activity, different factors should be taken into account to design tagged molecules, such as the introduction of a biotin moiety into the parent molecule that could result in steric hindrances of its interaction with certain targets (site of protein recognition), the preferential solubility in aqueous media, the presence of intact functional groups that are involved in the protein conjugation, and the stability of resulting biotinylated drug coupled to protein after conjugation. In search of all these aspects is a challenge in the case of CLV, especially due to its complex reactivity and instability after β-lactam opening (Finn et al., 1984; Martin et al., 1989; Baggaley et al., 1997; Brethauer et al., 2008). This probably has hampered any success in the detection of specific IgE antibodies through immunoassays as well as the production of antibodies against CLV.

CLV was successfully labeled with a biotin moiety in the carboxylic group at C3, through two different approaches that keep the β-lactam ring intact. Direct coupling rendered CLV-B, which was not soluble in water, needing proportions of DMSO to solubilize in aqueous media for performing protein incubation experiments. An improved design consisted in CLV-TEG-B, which included an extending TEG linker between CLV and the biotin moiety. Such hydrophilic linker increases the hydrophilicity of the compound, which provides solubility in aqueous media, besides the flexibility and length of the spacer would allow enough distance between CLV molecule and the biotin tag to potentially result in less steric hindrance interactions, both in the protein-CLV conjugation process, and making biotin moiety more available to interaction with streptavidin, leading to higher detection efficiency. Due to these advantages we focused mainly on CLV-TEG-B conjugation studies.

**FIGURE 6:**
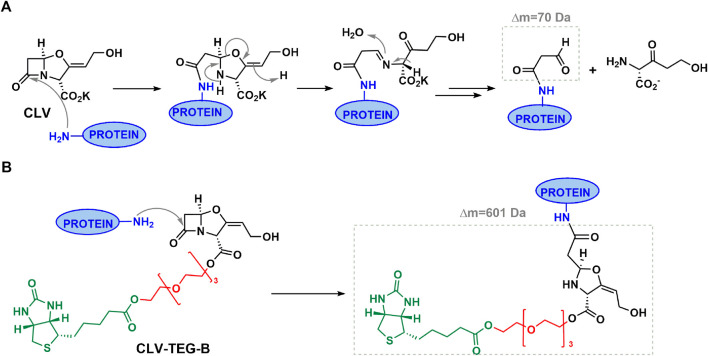
Hypothesized mechanisms for protein conjugation with clavulanic acid (CLV) derivatives. **(A)** Proposed reaction mechanism for covalent binding of CLV to proteins leading to an unstable structure that can degrade through different pathways resulting in different determinants. Represented only the structure of identified potential antigenic determinant (N -protein, 3-oxopropanamide). **(B)** Proposed reaction mechanism for binding of CLV-tetraethylenglycol-Biotin to proteins leading to a stable conjugate structure. Theorycal increment of mass added to protein is indicated in each case (see text for details).

Since CLV degrades at basic pH (Martin et al., 1989), physiological pH conditions were used for the biotinylated derivative conjugation as previously optimized for CLV (Barbero et al., 2019). This differs from optimized AX or other penicillins conjugations which have been reported to be performed in basic pH and which ensure lysine amino group deprotonation and favor nucleophilic attack to β-lactam carbonyl (Ariza et al., 2012; Pajares et al., 2020). Here we observed that CLV is very stable at physiological pH whereas 50% of CLV-TEG-B suffers hydrolysis in its β-lactam at 16 h, which would still permit 50% of CLV-TEG-B to conjugate during incubation time. We also have observed that both CLV and CLV-TEG-B display reactivity toward simple amines, through β-lactam ring opening. In spite of their similar reactivity in presence of nitrogen nucleophiles, they differ in terms of stability subsequent to conjugation. After amide formation the resulting conjugate for CLV breaks down into multiple compounds, whereas the conjugate formed with CLV-TEG-B does not suffer degradation in its biotinylated drug linked (Figure 6). Similar reactivity behavior was desired for both, parent and biotinylated drug, in order the tagged drug can emulate conjugation process occurring with the native drug (Ariza et al., 2014; Gonzalez-Morena et al., 2016). Nevertheless, the stability of the conjugated biotinylated analogue was a critical aspect for keeping the biotinylated moiety attached to the drug, and therefore their usefulness for detecting haptenated proteins.

Because published data identifies HSA as the main target for haptenation with drugs, we used this protein as a model for our study (Ariza et al., 2014). We observed that addition of CLV-TEG-B to HSA takes place *in vitro*, as deduced by MALDI-TOF MS, and that this modification is concentration-dependent, consistent with recent studies involving CLV (Meng et al., 2016; Barbero et al., 2019). These results are in agreement with the SDS-PAGE analysis of these conjugates, that showed that the extent of biotinylation was concentration-dependent of CLV-TEG-B and CLV-B used during incubation. MALDI-TOF MS analysis provided not a single mass but a molecular weight distribution containing protein species modified to a different extent, including unmodified protein, thus yielding only an estimate of the mass increment that may be related to the degree of haptenation. Moreover, MS comparison between conjugates with CLV-TEG-B and CLV (Barbero et al., 2019), and taken into account that the molecular weight of identified determinants of native CLV is 70 Da, indicates that haptenation taking place with CLV-TEG-B occurs in a lower extent than with CLV, which could be explained by the steric hindrances of the biotinylated moiety. The identification of HSA sites modified by CLV-TEG-B was only carried out for the conjugate formed using 1:10 protein:drug ratio, as performed previously with the native CLV (Barbero et al., 2019), although this concentration is higher than that of the drug *in vivo* under therapeutic conditions (Nagarajan et al., 2013). In the case of the biotinylated CLV, to get an exact mass increment of the fragment that links to the protein, enrichment of the biotinylated fraction was required to get any result by LC-MS/MS. We searched a 601.2 Da mass increase, related to the incorporation of a 602.2 Da with loss of the hydrogen atom of the HSA lysine fragment, compatible with the haptenation by one CLV-TEG-B thorough β-lactam ring opening without any other fragmentation. However, only a mass increment of 600.2 was found in the ^404–430^HSA peptide. An identical mass increment was found in a CLV-TEG-B treated-HSA peptide, used in a controlled experiment as a simpler model, which confirms that CLV-TEG-B adds to the protein with a 600.2 Da mass addition, although a low level of haptenation was obtained and the attached structure could not be elucidated. This ^182–195^HSA peptide has been previously reported to be modified *in vitro* by CLV-derived structures, consisting of *N*-protein, 3-oxopropanamide identified determinant of CLV (Barbero et al., 2019) or others bearing higher molecular weight (Meng et al., 2016), and by a series of penicillins, such as benzylpenicillin (Meng et al., 2011), AX (Garzon et al., 2014), flucloxacillin (Jenkins et al., 2009), and piperacillin (Whitaker et al., 2011).

Besides lysine residues, the modified ^404–430^HSA peptide contains other amino acid nucleophiles as arginine, which could be modified by acylation of the β-lactam. For instance, HSA haptenation with native CLV has been reported to occur also through histidine residues that are modified with the open β-lactam of the complete CLV molecule or with its pyrazine metabolite (Meng et al., 2016). However, modification of residues different from lysine was not observed in our previous study with native CLV (Barbero et al., 2019). In the present study, the modified ^404–430^HSA peptide seems to be adducted on the ^410–419^HSA sequence (RYTKKVPQVS). From these residues, we assume lysine as the residue with the highest reactivity toward this compound, due to its lower pka and previous literature regarding CLV protein binding (Meng et al., 2016; Barbero et al., 2019); however, at this point, there is no experimental evidence about which aminoacid forms the adduct with CLV-TEG-B. In any case, the modified peptide with CLV-TEG-B differ from those modified by original CLV, attached as 70 Da antigenic determinant, observed at two different residues, Lys 195 and Lys 475 (Barbero et al., 2019). This was somewhat expected, and could be explained by the presence of the biotin moiety that may impose steric impediments for binding to some targets or it may shield part of the molecule (Ariza et al., 2014). In spite of this, both compounds seem to compete for binding to proteins. This is in agreement with a previous study suggesting that AX and its biotinylated derivative could bind to protein common sites (Ariza et al., 2014). Besides competition for common site binding, other interferences in the protein could explain this behavior such as a change in the conformation structure of the protein and a binding/addition to closed sites that involves changes in steric and electronic effects of target lysine residues.

The chance of using biotinylated derivatives of CLV for the identification of modified serum proteins was studied by SDS-PAGE and blot followed by biotin detection since biotinylated CLV could be detected with a high sensibility using streptavidin conjugated with peroxidase. The analysis of HSA modification by biotinylated-CLV showed that protein extent modification was drug concentration-dependent. Importantly, the biotin moiety remained linked to the protein after conjugation to provide detection, and the method showed a great sensitivity for this application. Moreover, a semiquantitative estimation of biotinylation indicated that at 1:100 protein/drug molar ratio, only a 9% of the protein would form conjugate, if only one site were modified. This confirm the remarkable sensitivity of the method and may suggest a low protein modification extension, in agreement with the required enrichment of biotinylated fractions for high resolution MS techniques.

Serum proteins identified as candidate targets of CLV-TEG-B by 2D-electrophoresis and mass spectrometry were HSA, haptoglobin, and immunoglobulin heavy and light chains. In addition, the haptenation of transferrin could be proposed on the bases of its molecular weight and isoelectric point in 2D gels. This finding represents a great progress in our understanding of the mechanisms driving CLV allergy. Furthermore, these CLV derivatives could be useful for complex systems study, in which modified proteins could be purified with streptavidin columns. Previous studies of protein haptenation by AX have allowed to confirm the results obtained with biotinylated drug with those obtained with the native drug. By assaying serum protein conjugates with AX or biotinylated AX by 1D and 2D-electrophoresis and following Western blot using anti-AX antibodies for the AX detection or streptavidin for the biotin detection, it was observed that both compounds bind to the same serum targets (HSA, transferrin and IgE light and heavy chains), with the only difference of a weak binding to haptoglobin, which is undetectable using immunological AX detection, but is detectable with biotinylated AX due to the higher sensitivity of this method (Ariza et al., 2014; Martin-Serrano, 2018). The lack of antibodies against CLV impedes the immunological evaluation of protein-CLV conjugates and, therefore, we could not assess if the proteins haptenated by CLV are the same as the modified by its biotinylated derivative. However, this comparison study in the case of AX (Ariza et al., 2012; Ariza et al., 2014) could be somewhat extrapolated to the case of CLV.

## Conclusion

We have set up a model that may shed light into the process of protein haptenation by CLV through the use of highly sensitive approaches, such as labeling with biotinylated analogues, which allow the detection of its target serum proteins. These results strongly suggest that both, CLV and biotinylated CLV, are able to bind proteins through nucleophilic attack of the β-lactam carbonyl group by the protein amino nitrogen, process leading to the opening of the β-lactam ring. Unlike CLV protein conjugation, that promotes the decomposition of clavulanate fragment, the protein conjugates obtained with the CLV-TEG-B are stable enough to allow detection at great sensitivity. The results herein reported are of great interest since, for the first time, serum proteins that may act as carriers in allergic reactions to CLV are identified. Other alternative approaches for these studies are hampered by the complex reactivity of CLV and instability after conjugation. Further structural information on the binding sites on various targets would provide potential antigenic determinants to be used in diagnostic procedures and in studies on the mechanisms of CLV induced allergy.

## Data Availability Statement

The datasets presented in this study can be found in online repositories. The names of the repository/repositories and accession number(s) can be found in the article/Supplementary Material.

## Ethics Statement

The studies involving human participants were reviewed and approved by Comité de Ética de la Investigación Provincial. The patients/participants provided their written informed consent to participate in this study.

## Author Contributions

DP-S, EP-I, MJT and MIM conceived and designed the study. NB optimized synthetic approach and performed characterization of compounds. AM-S prepared compound in higher scale and performed reactivity studies. AS, JMM and FJSG performed proteomic experiments. AM-S, AA, DP-S and MIM analyzed all data, prepared figures, and wrote the manuscript, with input from MJT and EP-I.

## Funding

Work at MJT and MIM laboratory was supported by Instituto de Salud Carlos III (ISCIII) of MICINN (grants cofunded by ERDF: “Una manera de hacer Europa” (PI17/01237, PI18/00095, RETIC ARADYAL RD16/0006/0001 and Euronanomed Program AC19/00082), Miguel Servet I program (CP15/00103) and Sara Borrell program (CD17/00146)), Andalusian Regional Ministry of Health (PI-0179-2014, PE-0172-2018). Work at EP-I laboratory was supported by the Spanish Ministerio de Economía, Industria y Competitividad (CTQ 2016-75870-P), Ministerio de Ciencia y Educación (PID 2019-104293GB-I00), Ministerio de Ciencia e Innovación [Proyectos de I+D+I Programación Conjunta Internacional, EuroNanoMed 2019 (PCI 2019-111825-2)], ISCIII RETIC ARADYAL RD16/0006/0012 and Junta de Andalucía (UMA18-FEDERJA-007). Work at DP-S laboratory was supported by Grants from Agencia Estatal de Investigación, Ministerio de Ciencia e Innovación (MICINN, Spain) and European Regional Development Fund, SAF 2015-68590-R and RTI 2018-097624-B-I00, ISCIII RETIC ARADyAL RD16/0006/0021.

## Conflict of Interest

The authors declare that the research was conducted in the absence of any commercial or financial relationships that could be construed as a potential conflict of interest.
