# Supplementary material for: Biotin-Labelled Clavulanic Acid to Identify Proteins Target for Haptenation in Serum: Implications in Allergy Studies
Source: Front Pharmacol. 2020 Nov 18;11:594755. doi: 10.3389/fphar.2020.594755 (PMC7797785; doi:10.3389/fphar.2020.594755)
Supplement: Supplementary file 1 [file datasheet1.pdf]

Supplementary Material

Biotin-Labelled Clavulanic Acid to Identify Proteins Target for Haptenation in Serum: Implications in Allergy Studies

Ángela Martín-Serrano[1,2], Juan M. Gonzalez-Morena[3], Nekane Barbero[2,4], Adriana Ariza[1], Francisco J. Sánchez Gómez[3], Ezequiel Pérez-Inestrosa[2,4], Dolores Pérez-Sala[3], Maria J. Torres[1,2,5,6ꞁꞁ], María I. Montañez[1,2ꞁꞁ*]

[1]Allergy Research Group, Instituto de Investigación Biomédica de Málaga-IBIMA, Málaga, Spain

[2]Centro Andaluz de Nanomedicina y Biotecnología-BIONAND. Málaga, Spain

[3]Department of Structural and Chemical Biology, Centro de Investigaciones Biológicas Margarita Salas (CSIC), Madrid, Spain,

[4]Universidad de Málaga-IBIMA, Dpto. Química Orgánica. Málaga, Spain

[5]Allergy Unit, Hospital Regional Universitario de Málaga. Málaga, Spain

[6]Universidad de Málaga, Dpto. Medicina. Málaga, Spain

[ꞁꞁ]These authors contributed equally

*** Correspondence:**
Maria Isabel Montañez
maribel.montanez@ibima.eu

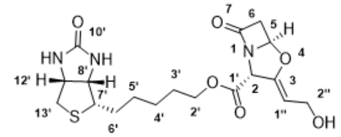

CLV-B

2-Biotin clavulanate (DMSO-d6)

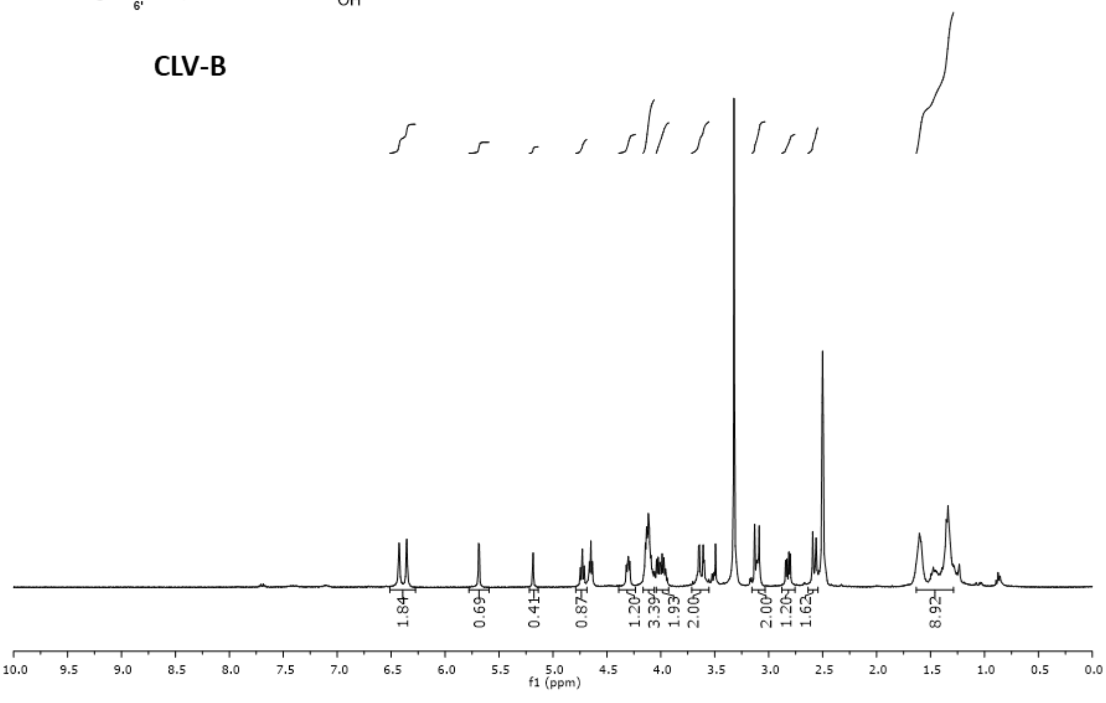

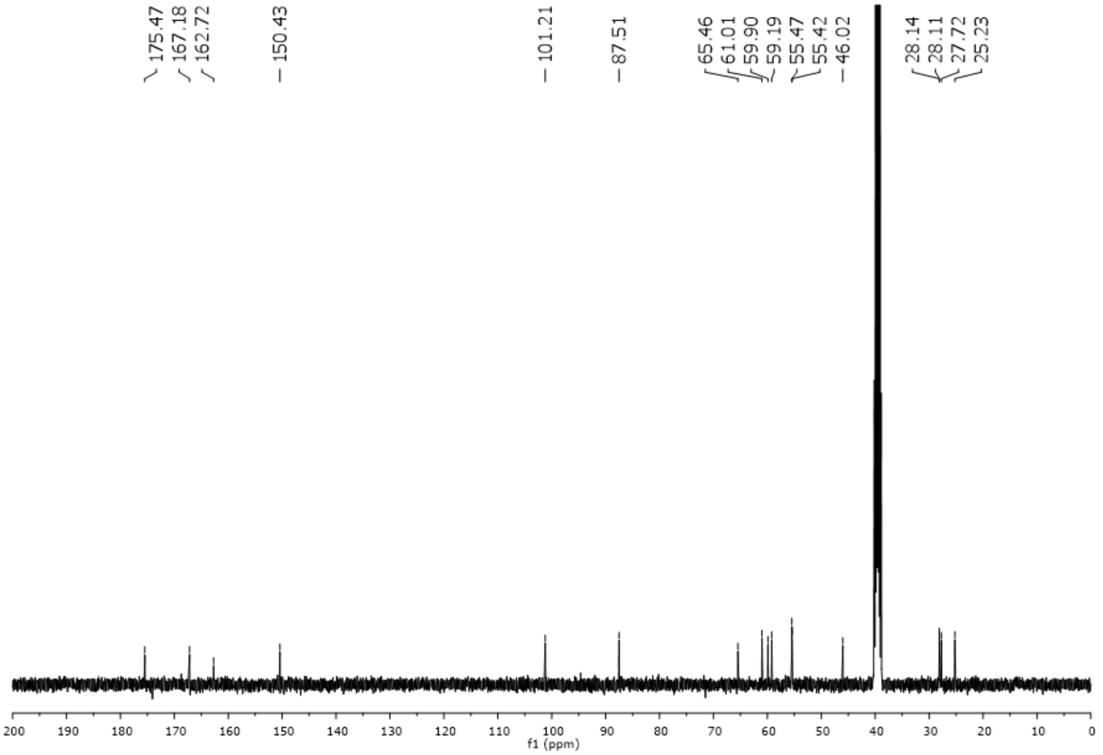

Supplementary Figure 1. ^{1}H-NMR and ^{13}C-NMR (DMSO) of CLV-B

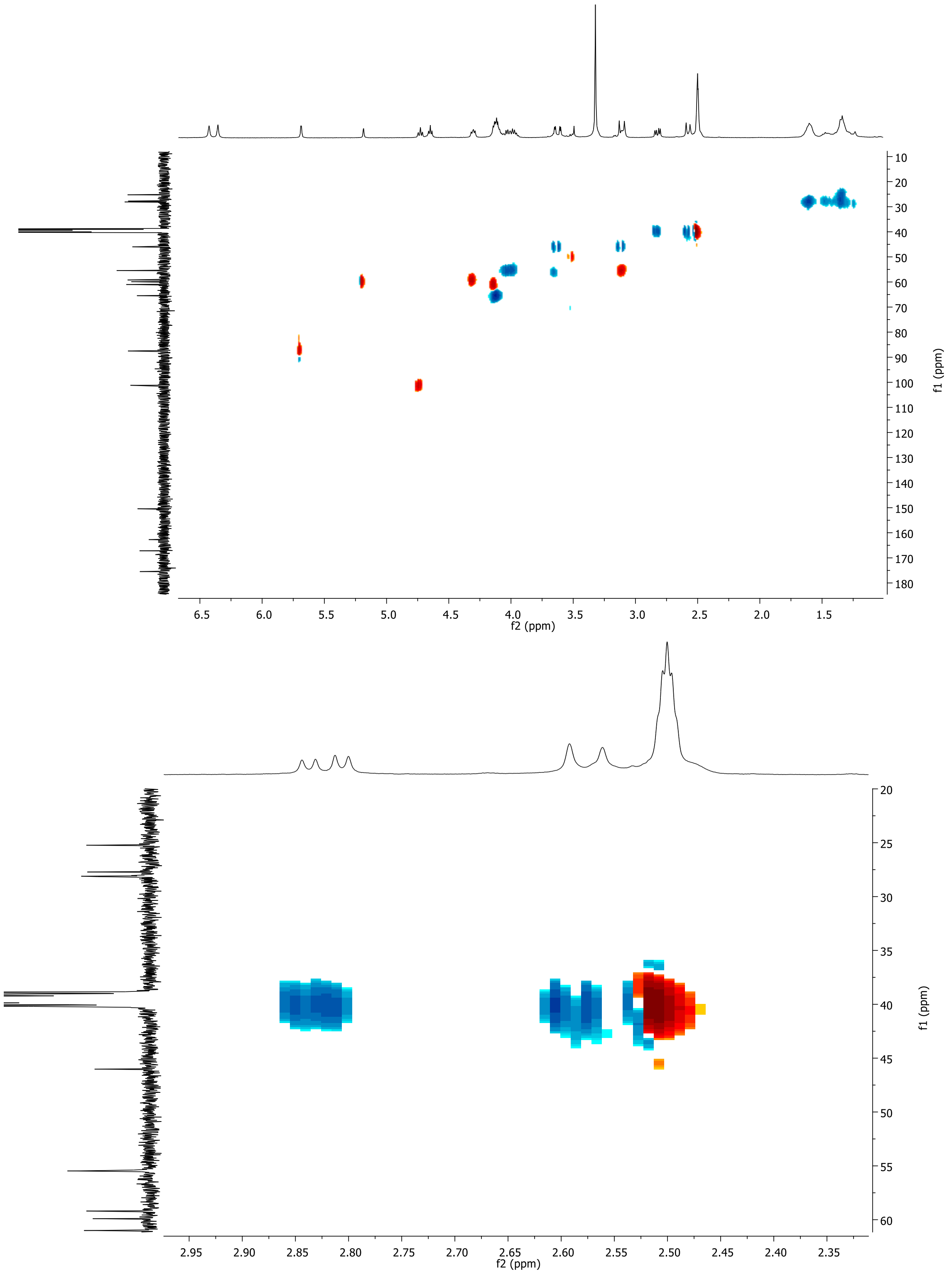

Supplementary Figure 2. HSQC (DMSO) of CLV-B

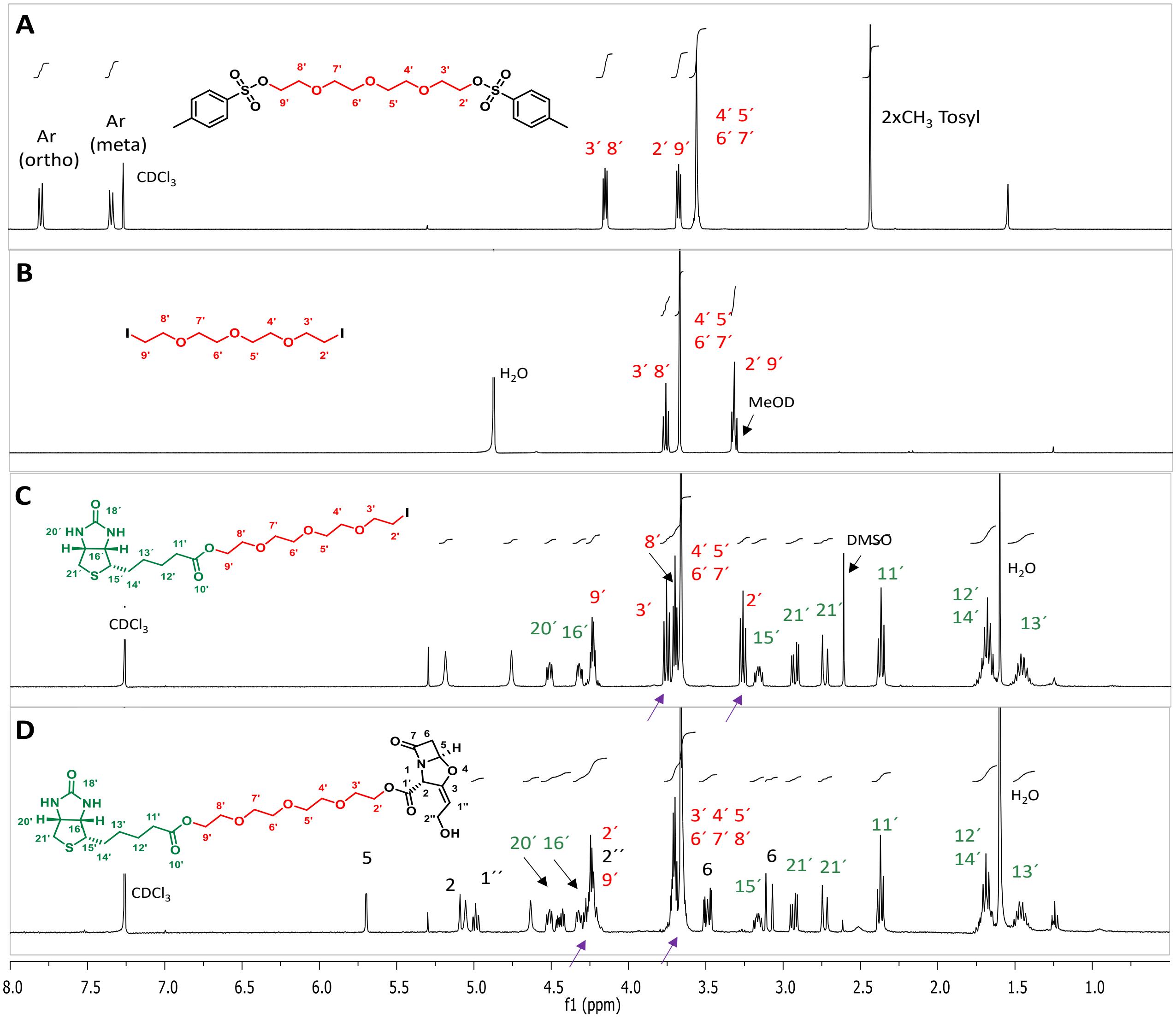

Supplementary Figure 3. ^{1}H-NMR monitoring of synthetic steps for preparing **CLV-TEG-B**. A) Ts-TEG-Ts. B) I-TEG-I. The disappearance of signals belonging to tosyl groups as well as the shift of the signals that correspond to methylene adjacent to tosyl groups (in A) to iodine groups (in B) confirm the reaction complexion. C) I-TEG-B. The signal corresponding to the methylene group bonded to iodine shifts from 3.25 ppm to 4.25 ppm, which confirms the coupling with biotin. D) CLV-TEG-B. The shift of signals corresponding to the methylene previously bonded to the iodine (2´´) to 4.25 ppm reflects the ester formation, which along with the apparition of CLV signals proved the reaction success.

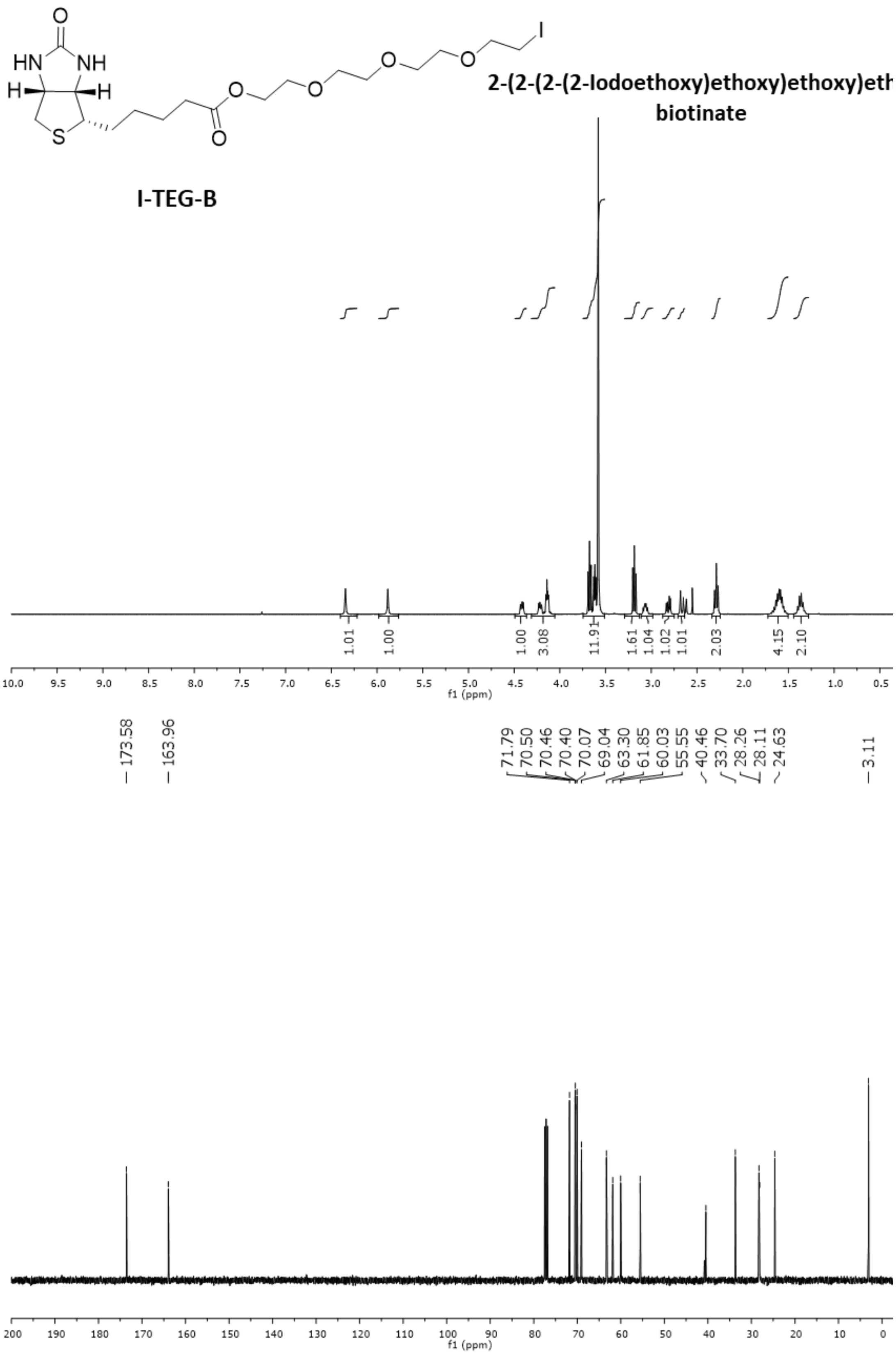

Supplementary Figure 4. ^{1}H-NMR and ^{13}C-NMR ($CDCl_3$) of I-TEG-B

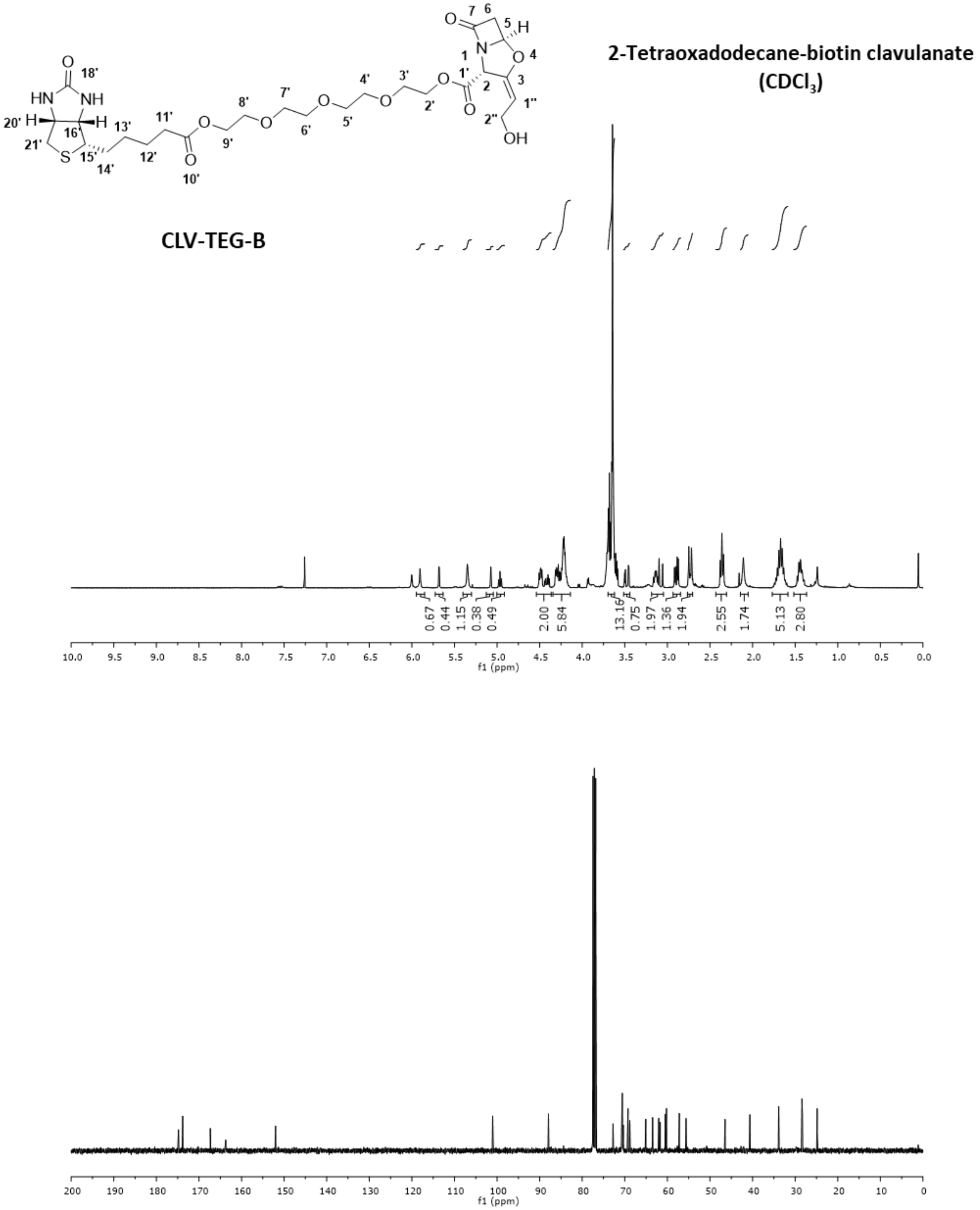

Supplementary Figure 5. ^{1}H-NMR and ^{13}C-NMR ($CDCl_3$) of CLV-TEG-B

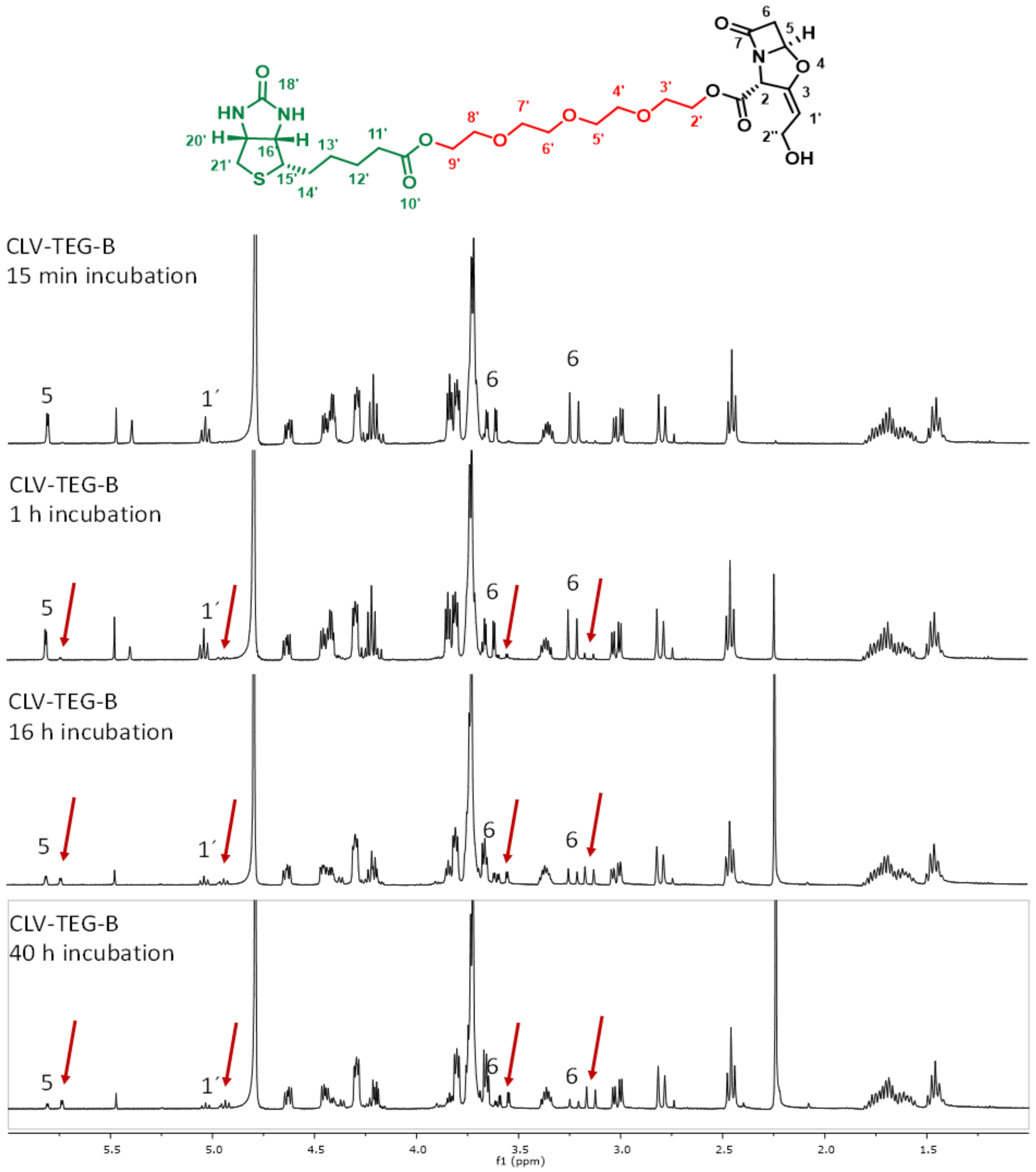

Supplementary Figure 6. ^{1}H-NMR spectra of CLV-TEG-B registered in deuterated PBS over time. Pointed with arrows the apparition of novel signals.

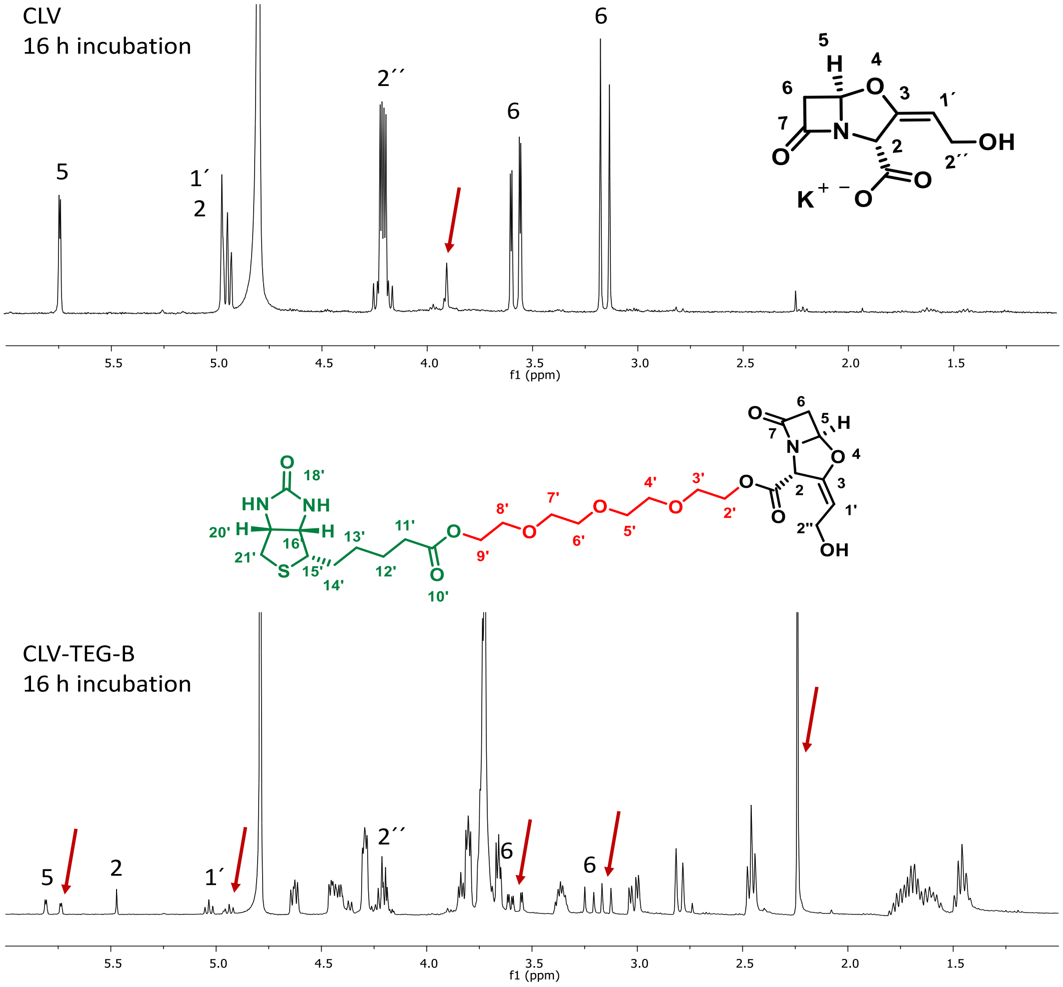

Supplementary Figure 7. ^{1}H-NMR spectra for stability comparison between CLV (top) and CLV-TEG-B (bottom) registered in deuterated PBS at the typical incubation time for preparing conjugates. Pointed with arrows new signals appeared as consequence of 16 h incubation.

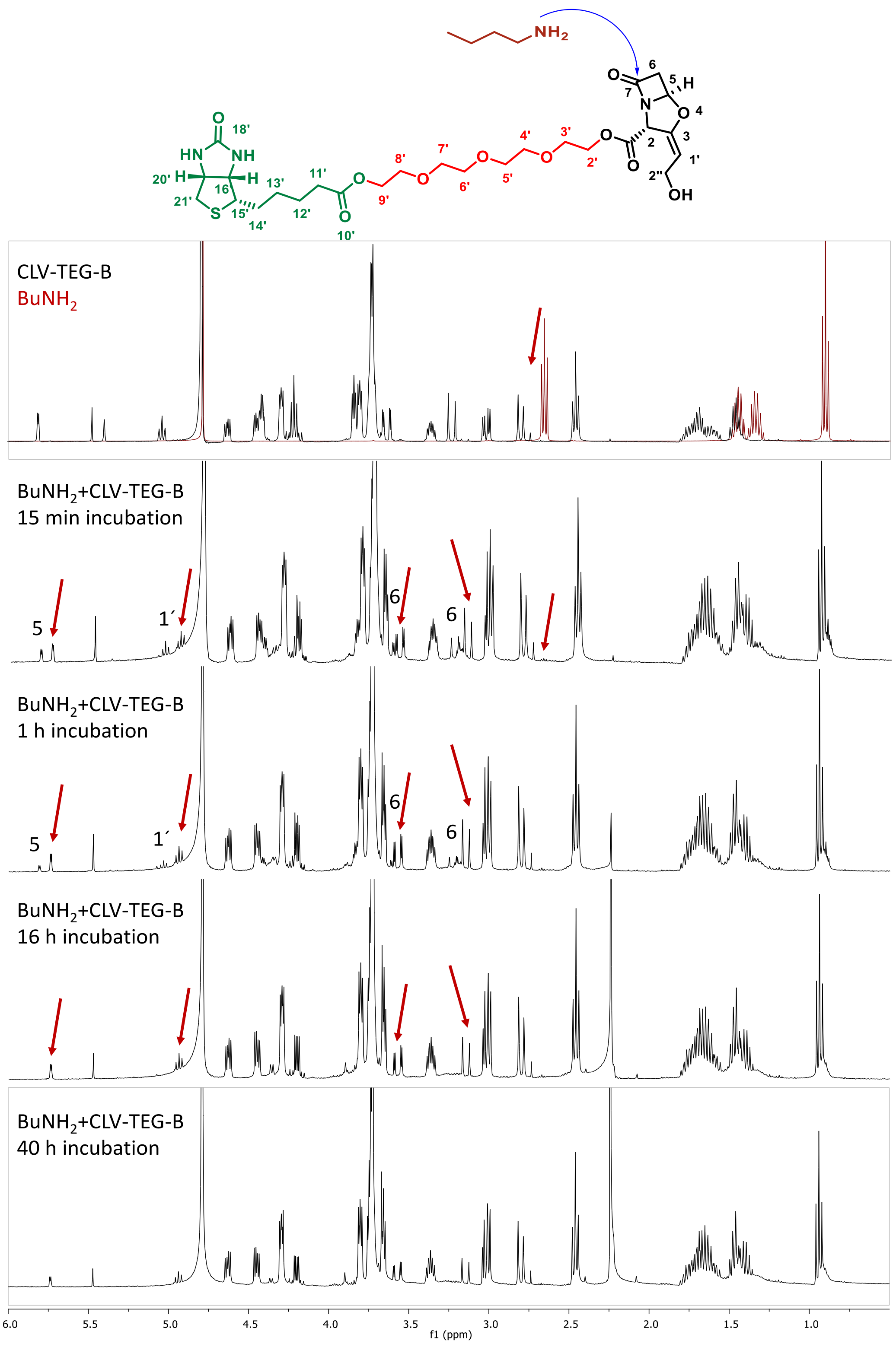

Supplementary Figure 8. Study of CLV-TEG-B reactivity with butylamine (1 equivalent) monitored by ^{1}H-NMR spectra registered in deuterated PBS at diferent times. Pointed with arrows changing signals. New signals at around 3.2 indicate amide formation consistent with disappearance of methylene groups directly bonded to amine groups of butylamine. After 16 h incubation the conjugate seems to be stable.

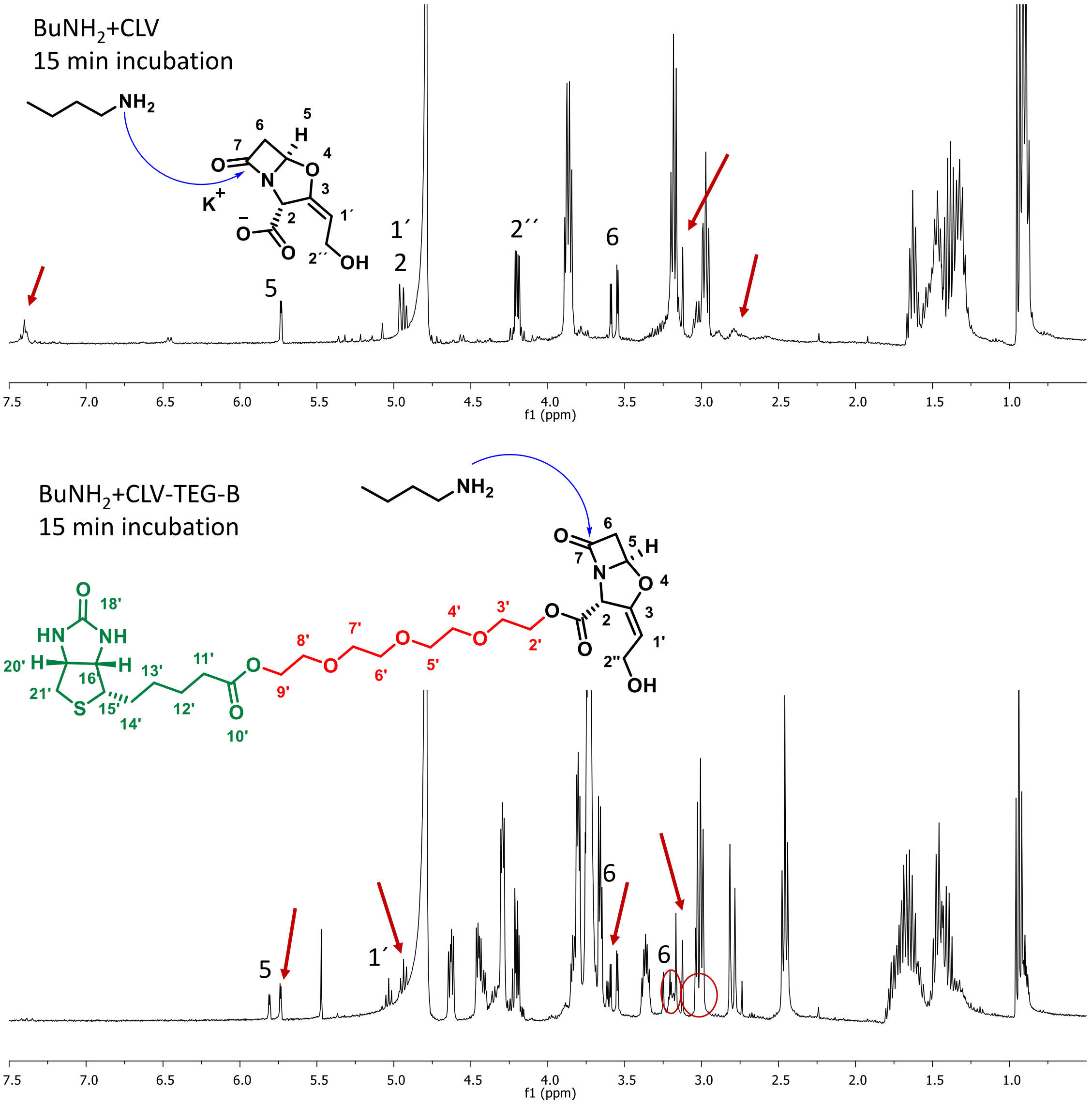

Supplementary Figure 9. ^{1}H-NMR spectra for reactivity comparison between CLV (top) and CLV-TEG-B (bottom) with butylamine (1 equivalent) after 15 minutes. Pointed with arrows and circles changing signals for comparison with native compound. New signals at around 3.2 indicate amide formation.

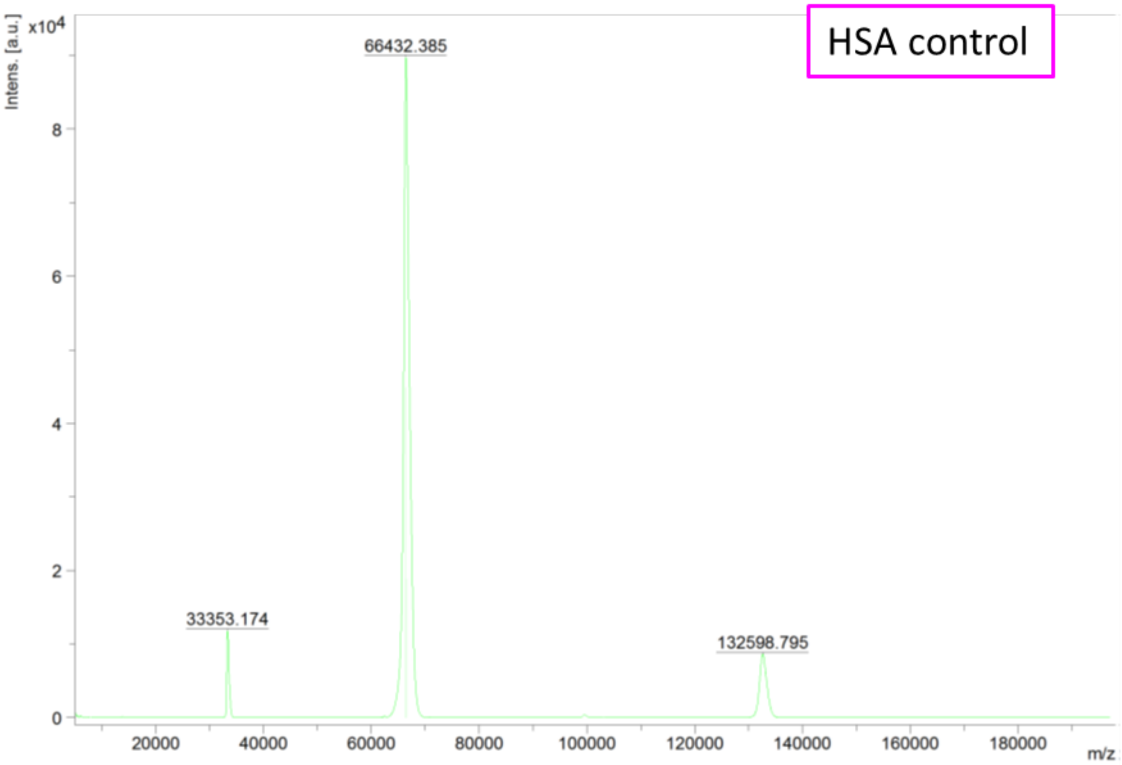

Supplementary Figure 10. **MALDI-TOF MS of HSA control.** HSA control was treated at same conditions that those described in Fig S11-S13, without drug incubation.

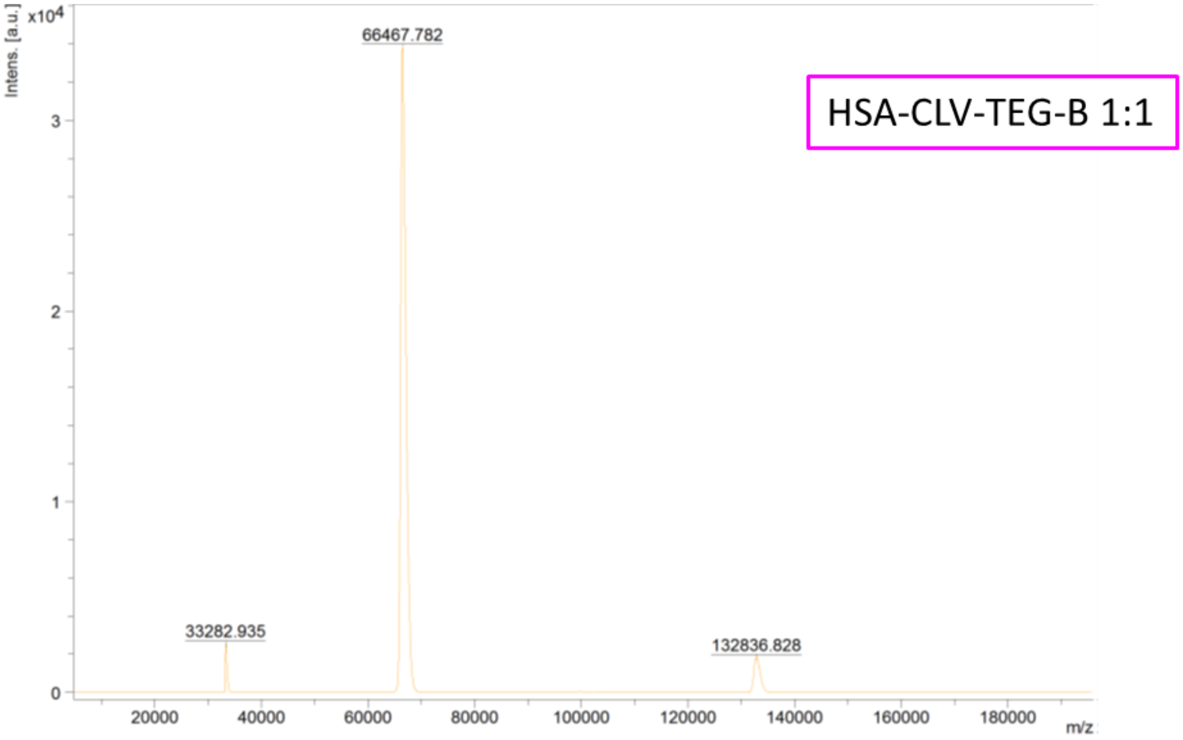

Supplementary Figure 11. MALDI-TOF MS of HSA-CLV-B 1:1. The conjugate was filtrated by dialysis filters and washed several times with bidistilled water and analized by MALDI-TOF MS.

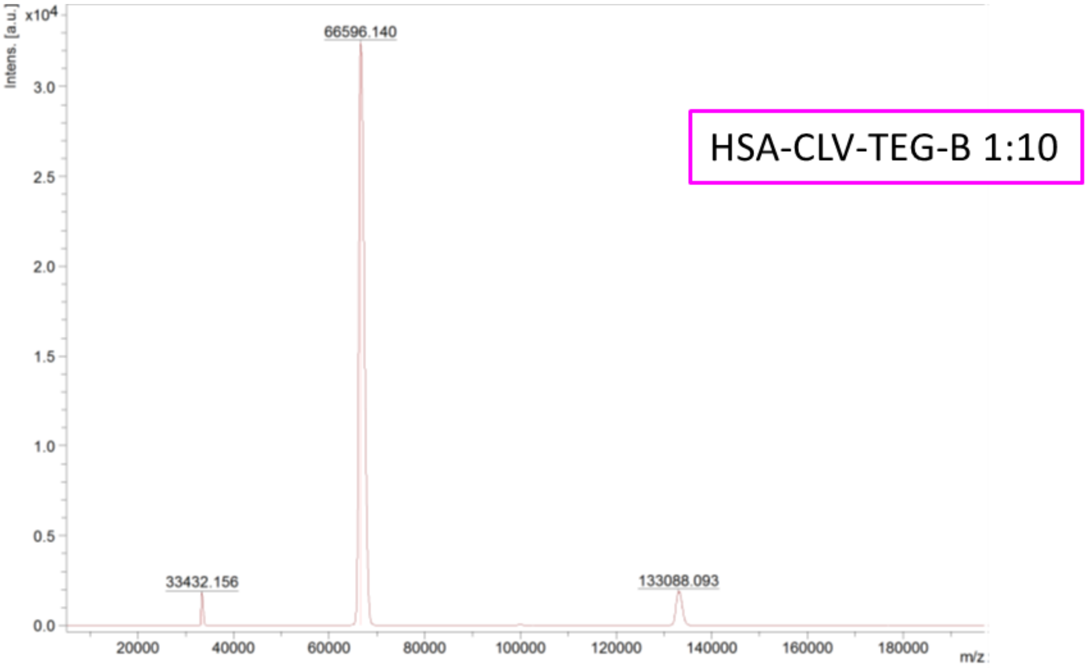

Supplementary Figure 12. MALDI-TOF MS of HSA-CLV-B 1:10. The conjugate was filtrated by dialysis filters and washed several times with bidistilled water and analised by MALDI-TOF MS.

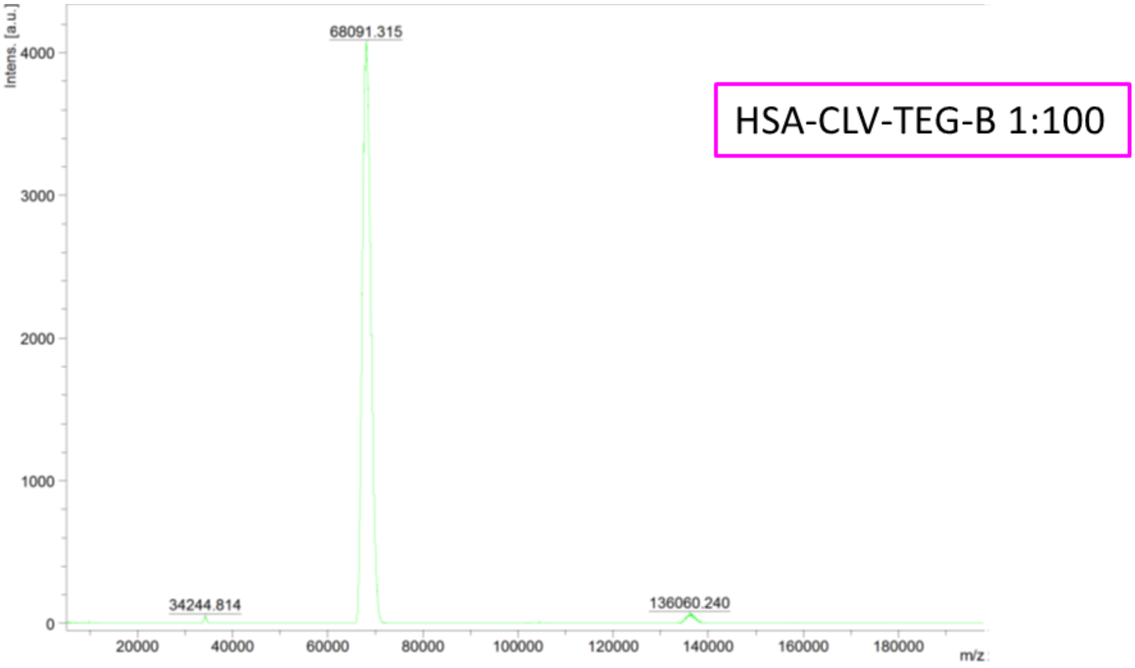

Supplementary Figure 13. **MALDI-TOF MS of HSA-CLV-B 1:100.** The conjugate was filtrated by dialysis filters and washed several times with bidistilled water and analysed by MALDI-TOF MS.

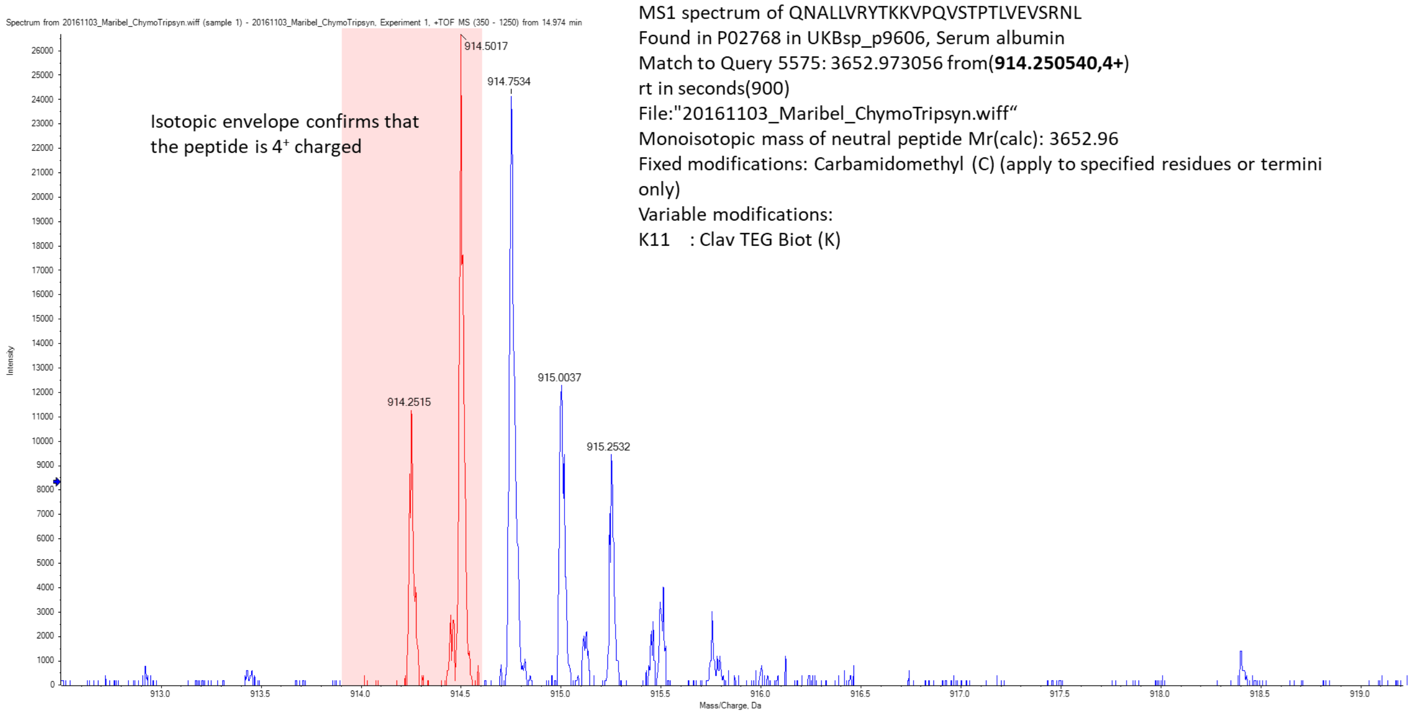

Supplementary Figure 14. Data of modified HSA peptide after conjugation with CLV-TEG-B. MS spectrum of QNALLVRYTKKVPQVSTPTLVEVSRNL molecular ion, peptide identified in vitro with a mass increment of 601.2 Da.

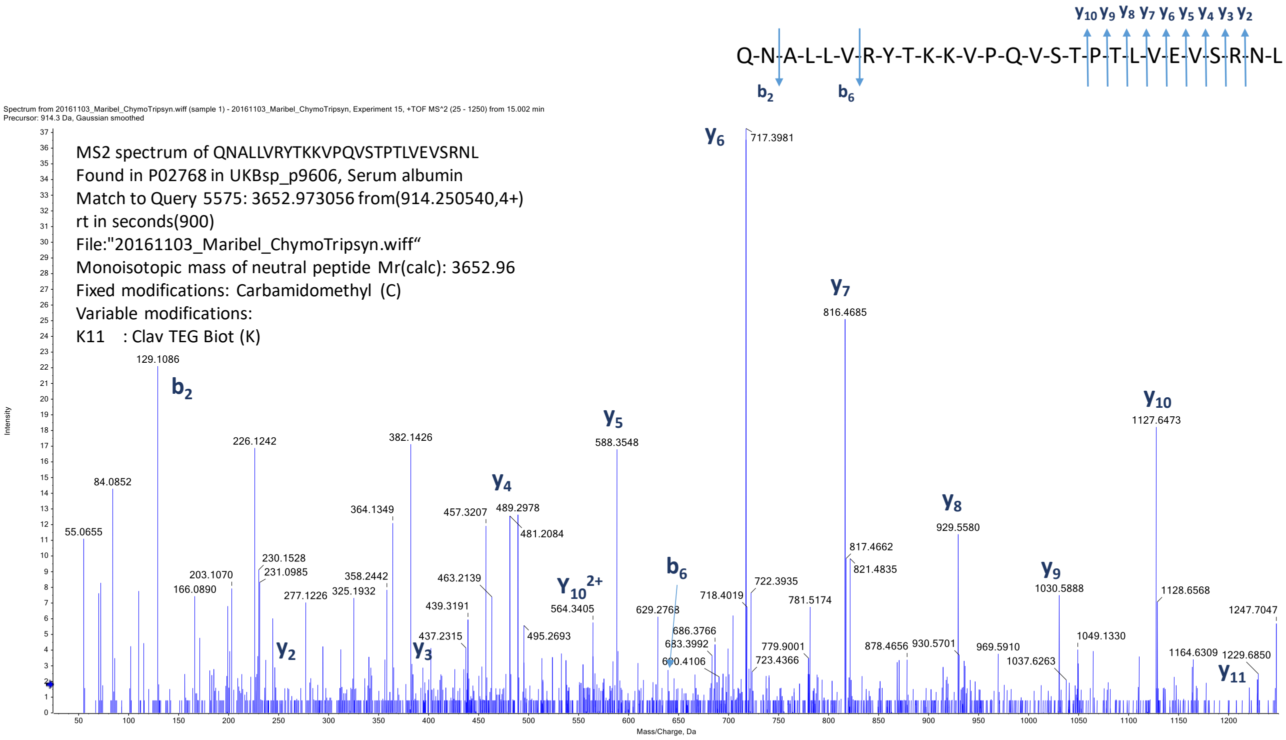

Supplementary Figure 15. Data of modified HSA peptide after conjugation with CLV-TEG-B. MS fragmentation spectrum of identified QNALLVRYTKKVPQVSTPTLVEVSRNL peptide.

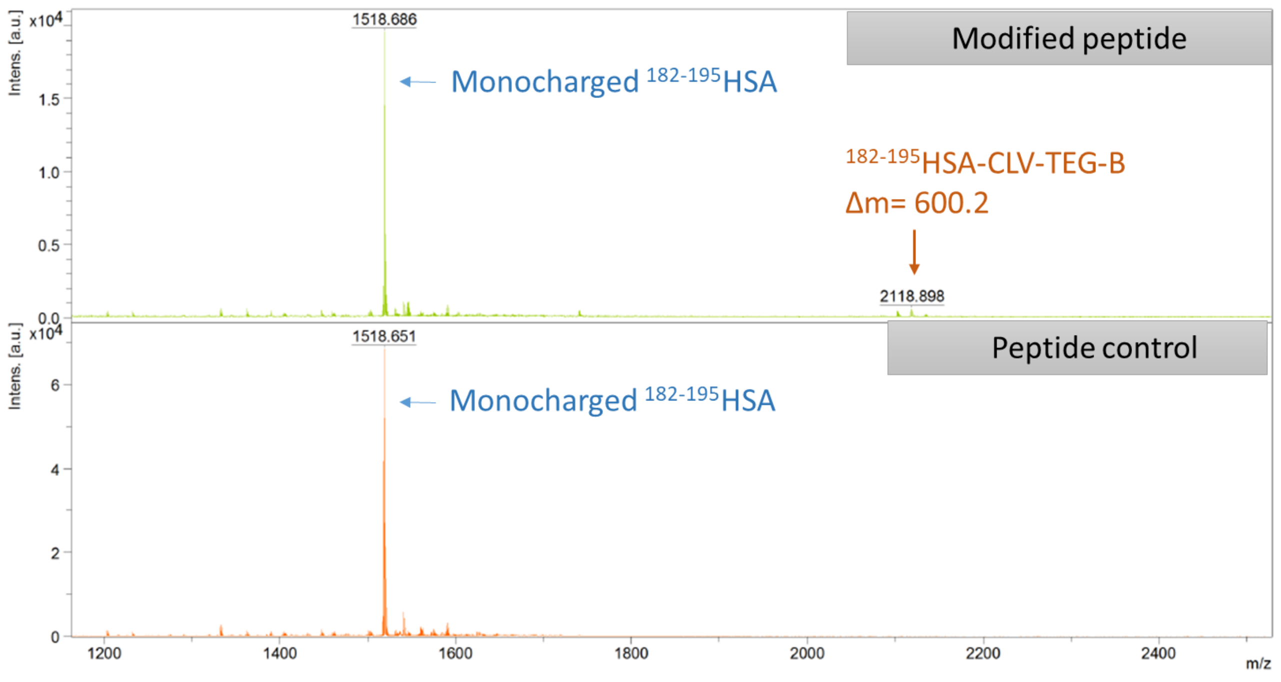

Supplementary Figure 16. MALDI-TOF MS spectra of $^{182\text{-}195}$HSA peptide conjugated to CLV-TEG-B. MALDI-TOF MS spectra of $^{182\text{-}195}$HSA peptide after conjugation with CLV-TEG-B (top) and control peptide, without conjugation (bottom).

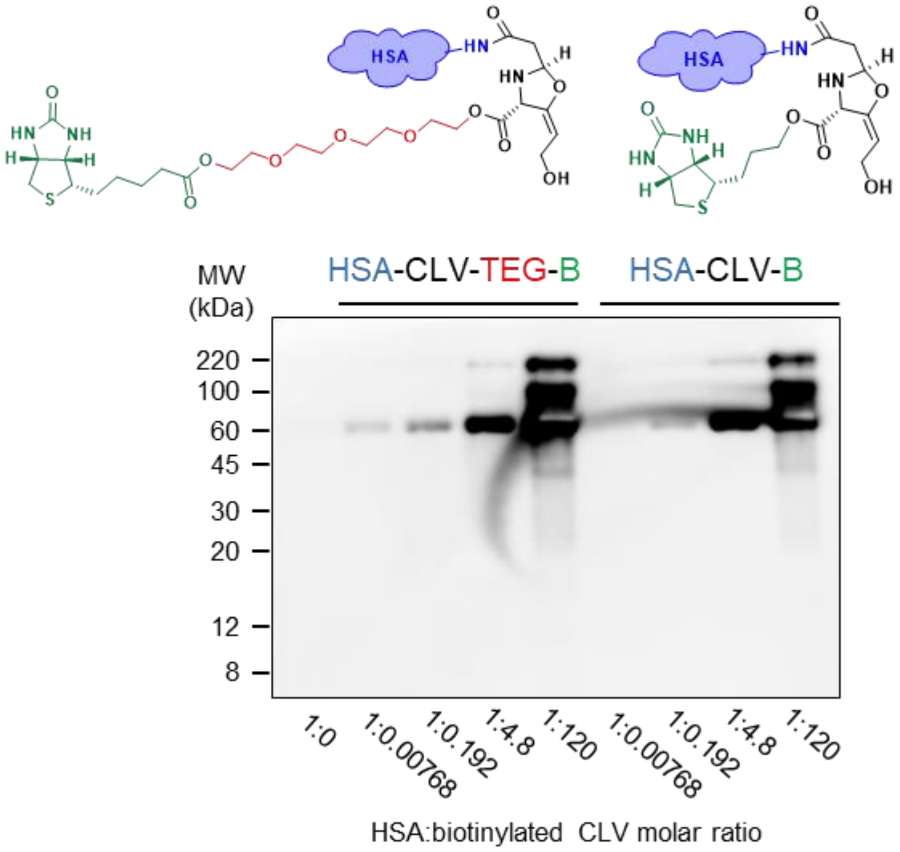

Supplementary Figure 17. **Detection of modification of HSA by biotinylated derivatives of CLV.** HSA was incubated in the presence of biotinylated derivatives of CLV for 16 h at 37˚C. HSA: biotinylated CLV molar ratios are showed in figure. 2 µg aliquots of resulting adducts were analyzed by SDS-PAGE and biotinylated CLV modification was detected by blot with streptavidin-HRP and ECL detection.

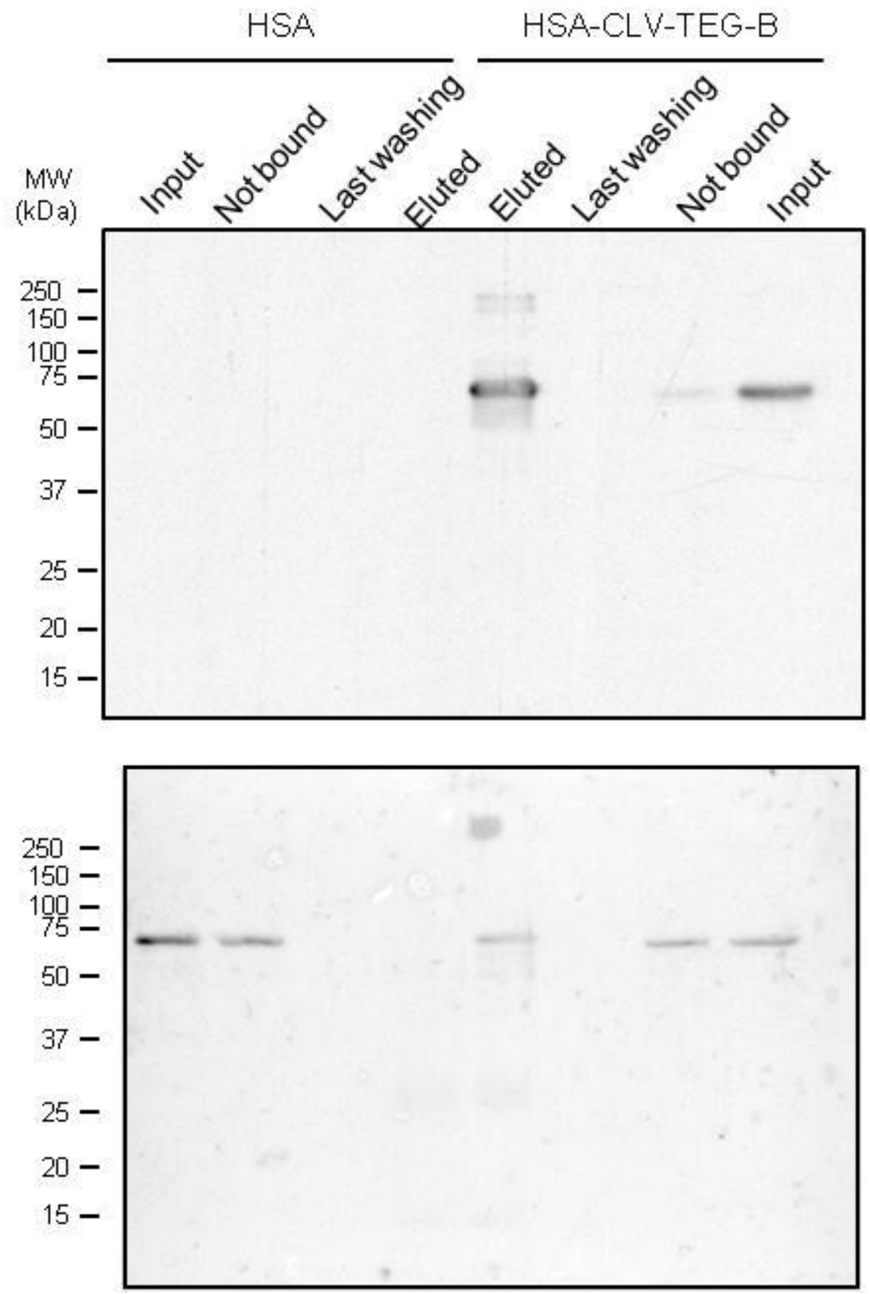

Supplementary Figure 18. **Detection of modification of HSA by biotinylated derivatives of CLV after enrichment of HSA-CLV-TEG-B fraction.** HSA-CLV-TEG-B fraction was obtained by affinity purification with neutravidin beads. Resulting samples were analyzed by SDS-PAGE and biotinylated CLV modification was detected by blot with streptavidin-HRP and ECL detection (top) and, after stripping with guanidine chloride, with Ponceau Red detection.
